# Ribosomal biogenesis regulator DIMT1 controls β-cell protein synthesis, mitochondrial function, and insulin secretion

**DOI:** 10.1016/j.jbc.2022.101692

**Published:** 2022-02-08

**Authors:** Gaurav Verma, Alexander Bowen, Sevda Gheibi, Alexander Hamilton, Sowndarya Muthukumar, Luis Rodrigo Cataldo, Olof Asplund, Jonathan Esguerra, Alexandros Karagiannopoulos, Claire Lyons, Elaine Cowan, Cristian Bellodi, Rashmi Prasad, Malin Fex, Hindrik Mulder

**Affiliations:** 1 Lund University Diabetes Centre, Lunds Universitet, Malmö, Sweden; 2 Division of Molecular Hematology, Department of Laboratory Medicine, Lund Stem Cell Center, Faculty of Medicine, Lund University, Lund, Sweden; 3 Unit of Islet Cell Exocytosis, Lund University Diabetes Centre, Malmö, Sweden; 4 Unit of Genomics, Diabetes and Endocrinology, Lund University Diabetes Centre, Malmö, Sweden

**Keywords:** DIMT1, methyltransferase, mitochondria, ribosomes, diabetes, COX1, cytochrome c oxidase I, DEG, differentially expressed gene, DIMT1, dimethyladenosine transferase 1, MAFA, MAF BZIP transcription factor A, mtDNA, mitochondrial DNA, ND1, NADH-ubiquinone oxidoreductase chain 1, NOB-1, NIN1 (RPN12) binding protein 1, OCR, oxygen consumption rate, PBS, phosphate-buffered saline, PDX-1, pancreatic and duodenal homeobox 1, PES-1, pescadillo ribosomal biogenesis factor 1, PLA, proximity ligation assay, rRNA, ribosomal RNA, SAB, secretion assay buffer, SNP, single-nucleotide polymorphism, T2D, type 2 diabetes, TFB1M, mitochondrial transcription factor B1, TMRM, tetramethyl-rhodamine methyl ester, tRNA, transfer RNA

## Abstract

We previously reported that loss of mitochondrial transcription factor B1 (TFB1M) leads to mitochondrial dysfunction and is involved in the pathogenesis of type 2 diabetes (T2D). Whether defects in ribosomal processing impact mitochondrial function and could play a pathogenetic role in β-cells and T2D is not known. To this end, we explored expression and the functional role of dimethyladenosine transferase 1 homolog (DIMT1), a homolog of TFB1M and a ribosomal RNA (rRNA) methyltransferase implicated in the control of rRNA. Expression of *DIMT1* was increased in human islets from T2D donors and correlated positively with expression of insulin mRNA, but negatively with insulin secretion. We show that silencing of *DIMT1* in insulin-secreting cells impacted mitochondrial function, leading to lower expression of mitochondrial OXPHOS proteins, reduced oxygen consumption rate, dissipated mitochondrial membrane potential, and a slower rate of ATP production. In addition, the rate of protein synthesis was retarded upon DIMT1 deficiency. Consequently, we found that DIMT1 deficiency led to perturbed insulin secretion in rodent cell lines and islets, as well as in a human β-cell line. We observed defects in rRNA processing and reduced interactions between NIN1 (RPN12) binding protein 1 homolog (NOB-1) and pescadillo ribosomal biogenesis factor 1 (PES-1), critical ribosomal subunit RNA proteins, the dysfunction of which may play a part in disturbing protein synthesis in β-cells. In conclusion, DIMT1 deficiency perturbs protein synthesis, resulting in mitochondrial dysfunction and disrupted insulin secretion, both potential pathogenetic processes in T2D.

Type 2 diabetes (T2D) is the result of dual defects of insulin secretion and action (1, 2). Pancreatic β-cells compensate for insulin resistance by secreting increased amounts of insulin. Over time, in genetically predisposed individuals, β-cells become exhausted and fail to maintain adequate insulin levels to overcome insulin resistance and maintain blood glucose. Hyperglycemia, insulin resistance, and hyperinsulinemia may also in themselves contribute to metabolic abnormalities (3, 4).

Insulin secretion from the β-cell is controlled by glucose metabolism (5, 6). Following a meal, glucose is taken up into β-cells by transporters, before being metabolized in glycolysis and the citric acid cycle. In these processes, the mitochondrion is a critical player, accounting for a major part of cellular metabolism (7). Importantly, mitochondrial metabolism produces ATP, which closes ATP-sensitive K^+^ channels, leading to depolarization of the plasma membrane (8). Subsequently, voltage-dependent Ca^2+^channels open, allowing cellular Ca^2+^ entry, which ultimately triggers insulin secretion (9, 10). Also, mitochondria generate a number of potential coupling factors, which amplify the secretion of insulin (6, 11). Therefore, β-cell function and insulin secretion are critically dependent on mitochondrial DNA (mtDNA) and protein expression (12, 13).

We have reported that a variant of the gene encoding transcription factor B1 mitochondrial (*TFB1M*) is associated with reduced insulin secretion, hyperglycemia, and future risk of T2D (12). TFB1M was initially considered to act as a transcription factor along with TFB2M, but more recent reports indicate its role as a methyltransferase (14, 15, 16). In mitochondria, TFB1M dimethylates two adjacent adenine residues in 12S rRNAs, conferring stability to the mitochondrial ribosome (17). Our studies showed that TFB1M deficiency leads to mitochondrial dysfunction and impaired insulin secretion resulting in diabetes (18). Despite this important role of RNA regulation and structure, very few studies have addressed the role of other methyltransferases in RNA methylation in β-cells (19). Therefore, we set out to examine the function of other methyltransferases that could be implicated in β-cell function and T2D.

To this end, we identified DIMT1 (dimethyladenosine transferase 1 homolog), which shows 50% homology with TFB1M and is expressed in human islets and β-cell lines. DIMT1 is known as a cytosolic rRNA methyltransferase, involved in ribosomal biogenesis, but its role in β-cells has not been explored. Using insulin-producing rat and human cell lines, as well as rat islets, we show that DIMT1 regulates cytosolic protein synthesis, impacting mitochondrial function and consequently insulin secretion. We have hereby identified a possible role of DIMT1 in β-cell ribosomal biogenesis and mitochondrial function.

## Results

### *DIMT1* expression in human islets, regulation in T2D, and correlations with glycemic traits

**Figure 1:**
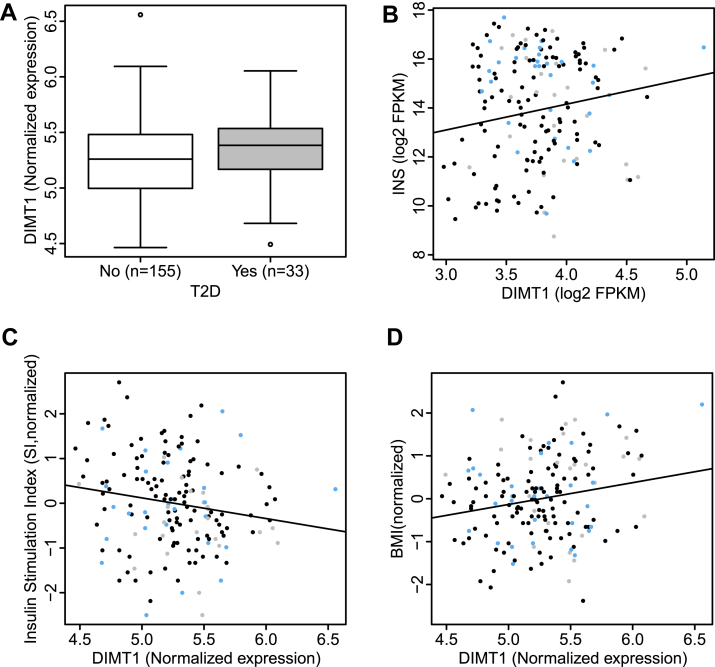
**DIMT1 expression in human islets and correlation with diabetic phenotype.** mRNA expression levels in islets from human donors were determined by RNA sequencing as described in (20). Difference in *DIMT1* gene expression between nondiabetic and T2D islet donors (*A*) (*p* = 0.041). Correlation of insulin gene (*INS*) and *DIMT1* gene expression (*B*) (*p* = 6.71 e-02). Insulin secretory index (SI) correlated with *DIMT1* gene expression (*C*) (*p* = 0.025). Correlation of body mass index (BMI) and *DIMT1* gene expression (*D*) (*p* = 0.0048). DIMT1, dimethyladenosine transferase 1; T2D, type 2 diabetes.

Given that DIMT1 is a homolog of TFB1M, a methyltransferase implicated in mitochondrial function and T2D, we mined our human islet RNA sequencing database (20). We found that *DIMT1* is expressed in human islets of Langerhans; its expression was significantly increased in T2D (Fig. 1*A*). We also found that *DIMT1* and insulin gene expression were positively correlated (Fig. 1*B*). There was a negative correlation between the insulin secretory index (SI), which reflects the fold response of insulin release from human donor islets in response to 16.7 mM glucose, and *DIMT1* expression (Fig. 1*C*). *DIMT1* was also found to be significantly correlated with BMI (Fig. 1*D*). In agreement with this finding, *DIMT1* expression correlated positively with HbA1c in donors of human islets (Fig. S1). Together, the data demonstrated that *DIMT1* expression reflects dysglycemia and T2D, and correlates with a number of glycemic traits. This may suggest a potential role of DIMT1 in regulation of human islet function and overall metabolic control.

Interestingly, several single-nucleotide polymorphisms (SNPs) mapping to the *DIMT1* locus, and nominally associated with *DIMT1* expression, were significantly associated with BMI and dietary intake. The same loci were also nominally associated with T2D risk, glycemic measures, such as 2-hour glucose and HbA1c, as well as fasting insulin levels (21, 22, 23, 24) (Table S1).

### Knockdown of *DIMT1* in INS-1832/13 and EndoC-βH1 cells and rat islets

**Figure 2:**
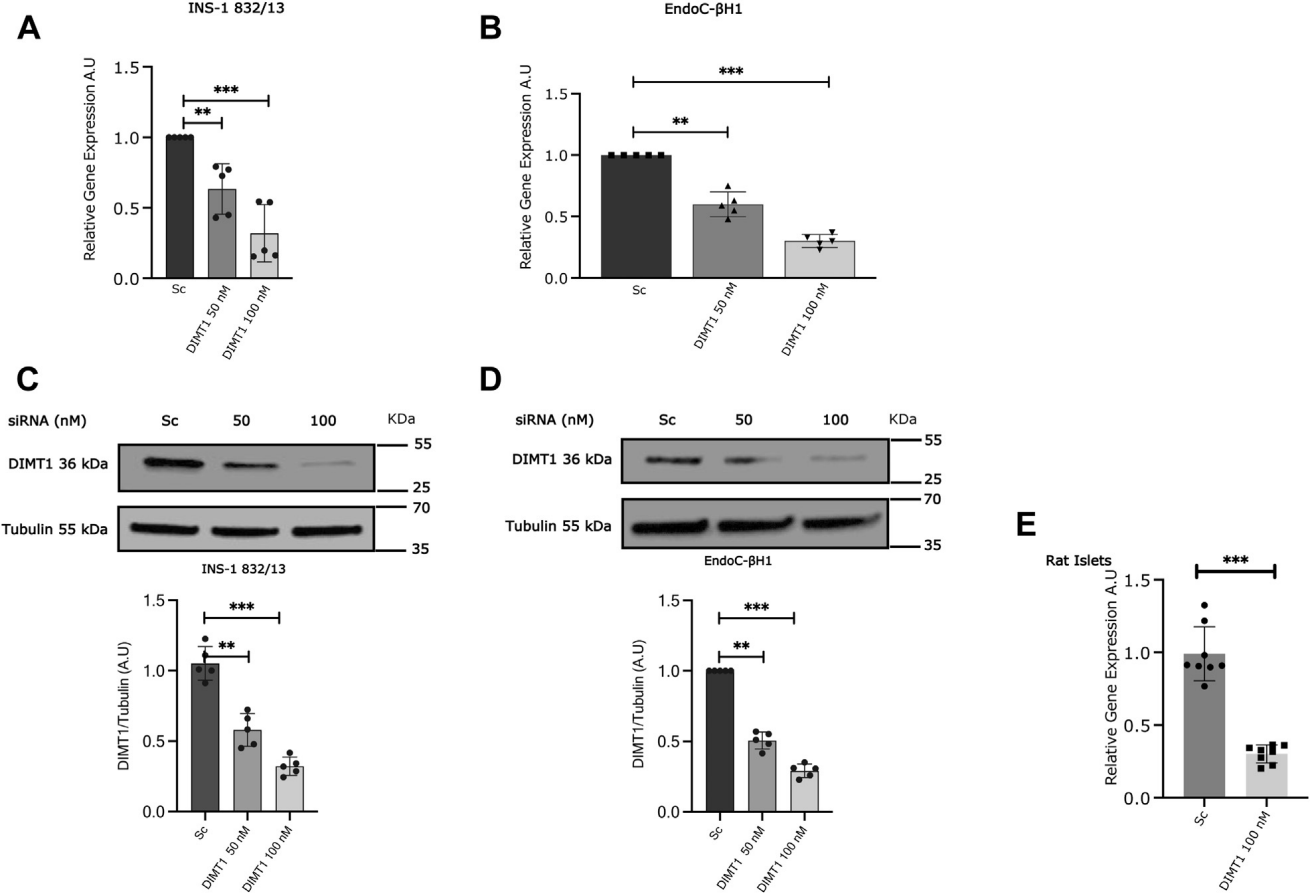
**DIMT1 knockdown in INS-1832/13 and EndoC-βH1 cells and rat islets.** Gene expression of *DIMT1* is shown in silenced INS-1832/13 (*A*) and EndoC-βH1 cells (*B*); cells treated with scramble siRNA served as control and actin mRNA expression as an internal control. DIMT1 silencing at the protein level was determined by Western blot in INS-1832/13 (*C*) and EndoC-βH1 cells (*D*) and tubulin was used as loading control; knockdown of DIMT1 in rat islets was analyzed by q-PCR as shown in (*E*). Data are expressed as mean ± SD (n = 5, EndoC-βH1, n = 5, INS-1832/13 and n = 4 in rat islets) and were statistically compared with paired Student’s *t* test; ∗∗*p* < 0.01, ∗∗∗*p* < 0.001. DIMT1, dimethyladenosine transferase 1

To elucidate the functional role of DIMT1 in islets, we chose a loss-of-function approach. We used siRNA targeting separate regions of *DIMT1* mRNA in INS-1832/13 and EndoC-βH1 cells and rat islets. Our knockdown experiments utilized two different siRNAs and a SMARTpool from Dharmacon. After verifying that these three siRNA approaches exhibited similar effects on *DIMT1* expression, we continued our experiments using 100 nM siRNA2, which most robustly silenced *DIMT1* without apparent toxicity (Fig. S1*B*). Figure 2, *A*–*D* shows the knockdown of *DIMT1* mRNA and DIMT1 protein, respectively, in the cell lines, and Figure 2*E* shows the *DIMT1* mRNA knockdown in rat islets. One-hundred nM siRNA effectively silenced mRNA and protein levels by ∼ 80% both in INS-1832/13 and EndoC-βH1 cells 72 h posttransfection; a similar level of *DIMT1* knockdown was observed in rat islets (∼70%; Fig. 2*E*). To corroborate our knockdown approach on a functional level, we also tested SMARTpool siRNA in one of our experiments that showed similar results as compared with the siRNA2 used throughout of the study (Fig. S1*C*).

### *DIMT1* knockdown and insulin secretion

**Figure 3:**
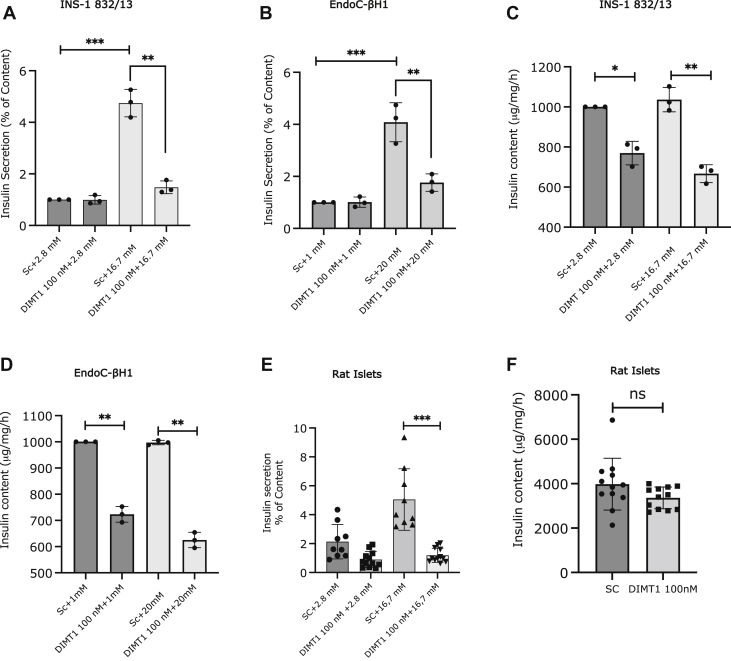
**DIMT1 deficiency and its impact on insulin secretion.** Insulin secretion, 72 h after of siRNA transfection at 2.8 and 16.7 mM glucose in INS-1 832/13 cells is shown in (*A*); insulin secretion at l and 20 mM glucose in EndoC-βH1 cells (*B*). Insulin content is shown in both cell types upon glucose stimulation with similar levels of knockdown (*C* and *D*). Insulin secretion (at 2.8 and 16.7 mM glucose) and content in rat islets (*E* and *F*); total cellular protein was used for normalization both for insulin secretion and content. Data are expressed as mean ± SD (n = 3, INS-1 832/13, n = 3, EndoC-βH1, n = 4, rat islets) and were statistically compared with paired Student’s *t* test; ∗*p* < 0.05, ∗∗*p* < 0.01, ∗∗∗*p* < 0.001. DIMT1, dimethyladenosine transferase 1.

Next, we investigated the effect of siRNA-mediated *DIMT1* knockdown on glucose-stimulated insulin secretion (GSIS) and insulin content in INS-1832/13 and EndoC-βH1 cells and rat islets. Stimulation with 16.7 mM glucose led to a fivefold increase in insulin secretion in INS-1832/13 cells; in EndoC-βH1 cells, 20 mM glucose increased insulin release by fourfold as compared with low glucose (Fig. 3, *A*–*D*). In contrast, *DIMT1* knockdown in either cell line resulted in nearly a threefold decrease in insulin release in response to high glucose (Fig. 3, *A*–*D*). A rise from low to high glucose concentrations did not significantly affect insulin content in either cell line. However, upon *DIMT1* knockdown, the insulin content was significantly reduced in both INS-1832/13 and EndoC-βH1 cells when compared with cells treated with scrambled siRNA (Fig. 3, *C* and *D*).

To replicate our results in primary cells, we determined insulin secretion and content in rat islets. We found a 2.5-fold increase in insulin secretion upon stimulation with 16.7 mM glucose in islets treated with scrambled siRNA; there was a threefold decrease in insulin release to 16.7 mM glucose upon DIMT1 deficiency (Fig. 3*E*). Our insulin secretion data in rat islets are thus in line with the data from the cultured cells. However, insulin content in rat islets upon *DIMT1* knockdown was not significantly reduced (Fig. 3*F*).

### DIMT1 and methylation of 18S rRNA in β-cells

**Figure 4:**
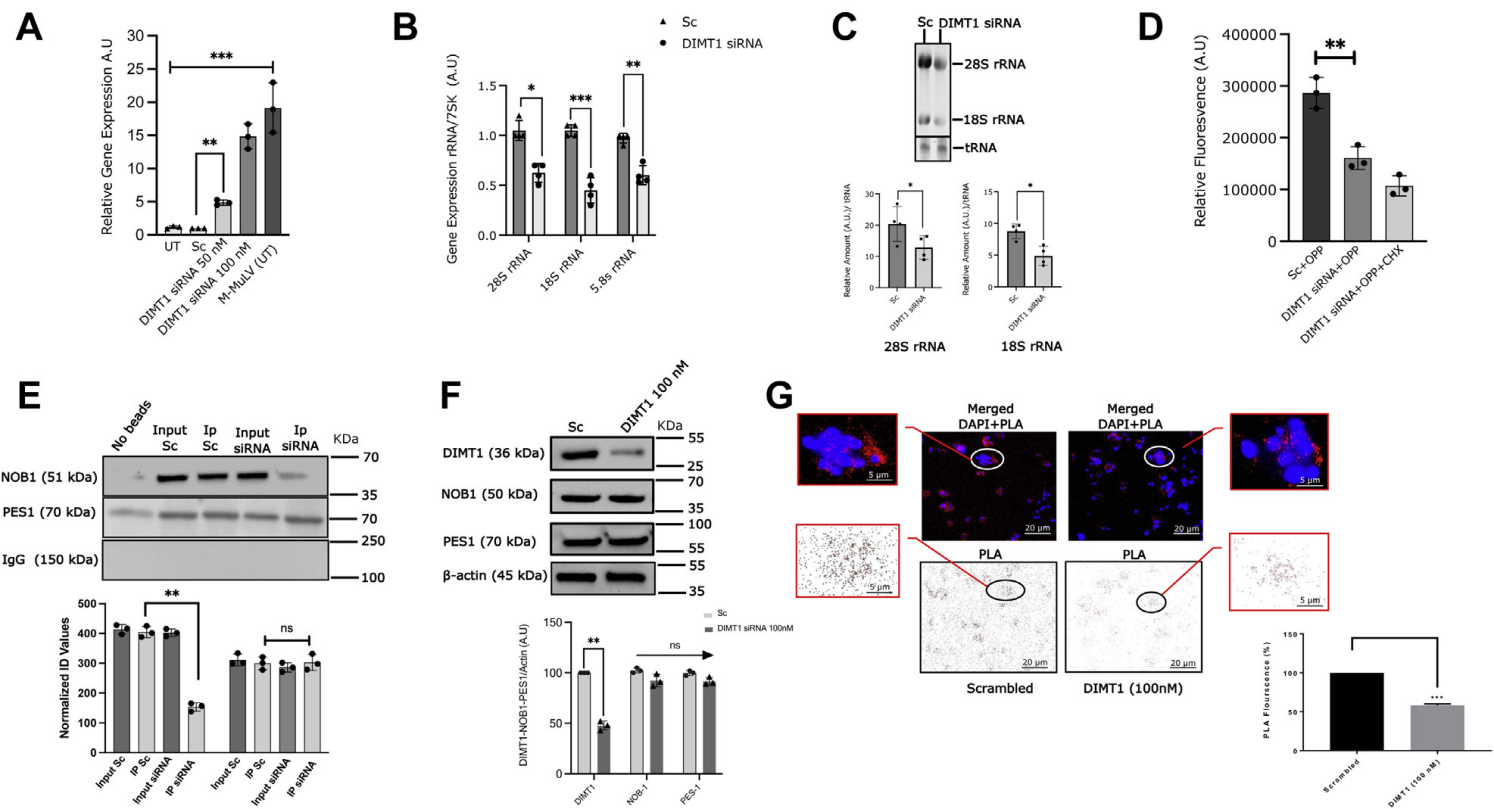
**The role of DIMT1 role in 18S methylation and cellular protein expression.** Quantification of 18S ribosomal rRNA methylation from total RNA was made by using the qRT-PCR gene expression method (*A*). The control EndoC-βH1 cells and cells treated with *DIMT1* siRNA are shown in the first 4 bars and the last bar represents the positive control, respectively. Data are expressed as mean ± SD (n = 4). Ribosomal RNA expression for 28S, 18S and 5.8S (*B*); expression of 28S, 18S and 5.8S rRNA in scramble (Sc)- and *DIMT1* siRNA-treated rat islets are shown (*B*). The average rRNA value was normalized by 7SK. Data are expressed as mean ± SD, (n = 4 in rat islets), and were statistically compared with paired Student’s *t* test; ∗*p* < 0.05, ∗∗*p* < 0.01, ∗∗∗*p* < 0.001. RNA Electrophoresis of 28S and 18S rRNA (*C*); for analysis of 28S and 18S rRNA, 2 μg of total RNA was resolved on denaturing agarose gels and the relative rRNA amount was normalized to total tRNA; the data are expressed as mean ± SD, (n = 4 in rat islets), and were statistically compared with paired Student’s *t* test; ∗*p* < 0.05. Protein synthesis detection using OPP (*D*); the first bar shows the Alexa 568 fluorescence intensity in control and the second and third bar shows the reduced OPP fluorescence in *DIMT1* siRNA- and cycloheximide (CHX)-treated cells, respectively. Data are expressed as mean ± SD (n = 3). Physical interaction of NOB1 and PES-1 (*E*) and their expression in whole-cell lysates with *DIMT1* siRNA (*F*). Western blot and immunoprecipitations are expressed as mean ± SD (n = 3). Proximity ligation assay (PLA; *G*) for NOB-1/PES-1 interaction is measured by *red dot* signals that represent the proximity of the NOB-1 and PES-1 proteins. DNA is stained with DAPI. Scale bar: 10 μM. Quantification of the PLA signals per cell after DIMT1 siRNA treatment is shown in the inserted graph (data are mean ± SD; n = 3); statistical comparisons between DIMT1-silenced cells and scramble control cells were made by Student’s *t* test; ∗∗*p* < 0.01, ∗∗∗*p* < 0.001. DIMT1, dimethyladenosine transferase 1; rRNA, ribosomal RNA

DIMT1 has been reported to be responsible for N^6^, N^6^ adenosine dimethylation at positions A_1850_ and A_1851_ in human 18S rRNA (25). To examine whether DIMT1 also serves as a dimethylase in human β-cells, we performed primer extension assays with primers flanking the methylation region in human 18S rRNA. As shown in Figure 4*A*, Hemo KlenTaq failed to transcribe total RNA isolated from untransfected EndoC-βH1 cells or cells treated with scrambled siRNA, indicating the presence of methyl groups bound to the target RNA sequence. In contrast, knockdown of *DIMT1* (50 and 100 nM) concentration-dependently increased amplification of the target RNA, indicating loss of methylation. Amplification with M-MuLV was not affected, substantiating our results. We conclude that DIMT1 specifically methylates 18S rRNA in EndoC-βH1 cells (Fig. 4*A*). The detailed principle of the method of primer extension is depicted in Fig. S2.

### DIMT1 deficiency, rRNA and protein synthesis in β-cells and rat islets

Ribosomes are required for protein synthesis in cells. To further elucidate the mechanism of reduced insulin secretion and content, we investigated ribosomes and quantified rRNA of the ribosomal subunits by real-time PCR and RNA electrophoresis in rat islets. Levels of the 60S rRNA, (28S and 5.8S) and 40S rRNA (18S) were found to be downregulated upon *DIMT1* deficiency (Fig. 4*B*). To substantiate our results, we ran RNA electrophoresis and found significant decreases in 28S and 18S rRNA expression in rat islets subjected to knockdown of *DIMT1* (Fig. 4*C*); these reductions may underlie the defective nature of the mature rRNA of both the ribosomal subunits. Our result indicates that the depletion of rRNA may compromise ribosomal biogenesis, affecting either subunit, and disturb rRNA processing leading to translational defects.

To examine whether reduced rRNA maturation impacts protein synthesis, we assayed protein synthesis in *DIMT1*-silenced EndoC-βH1 cells with modified puromycin (O-propargyl-puromycin; OPP). We observed that OPP addition led to less total protein synthesis upon *DIMT1* knockdown, as compared with the scramble control. Cells treated with the scrambled siRNA and OPP (SC+OPP) showed a significant increase in OPP fluorescence, reflecting increased global protein synthesis rates in these cells (Fig. 4*D*). In contrast, DIMT1-deficient cells showed a decreased rate of protein synthesis (Fig. 4*D*). The addition of cycloheximide showed no further significant reduction of OPP labeling, confirming the reduced protein synthesis rates upon DIMT1 deficiency (Fig. 4*D*). There were no effects on cell viability in the cells treated with *DIMT1* siRNA and the scramble siRNA, as measured by a trypan blue assay (Fig. S1*C*). Of note, overall protein synthesis was examined, including mitochondrial protein synthesis. At this point, it was not possible to distinguish between cellular or mitochondrial protein synthesis rates. Nevertheless, we conclude that DIMT1 plays a role in ribosomal biogenesis and β-cell protein synthesis.

### DIMT1 and rRNA processing in β-cells

So far, we observed that DIMT1 deficiency leads to immature rRNA production, impairs protein synthesis, insulin secretion and content, but the underlying molecular mechanism remained to be determined. Recent reports have suggested that DIMT1 and WBSCR22 methylate 18S rRNA, which is required for pre-rRNA processing reactions leading to stabilization of the ribosomal subunit (25). Therefore, we further investigated the role of DIMT1 in ribosomal biogenesis and protein synthesis. We used immunoprecipitation and a proximity ligation assay (PLA) to test whether DIMT1 deficiency perturbs ribosomal protein interaction in EndoC-βH1 cells.

To this end, we chose two critical proteins involved in 40S and 60S rRNA processing for analysis by immunoprecipitation (IP): NOB-1 (Nin1, binding protein), an endonuclease involved in the assembly of 40S rRNA, primarily required for cleavage of the 20S pre-rRNA to generate the mature 18S rRNA at the late maturation stage, and PES-1 (pescadillo ribosomal biogenesis factor 1), an essential ribosomal biogenesis factor of the 60S subunit. As shown in Figure 4*E*, an antibody to PES-1 was able to pull down NOB-1 protein in the EndoC-βH1 cell lysates both in scrambled controls and in *DIMT1*-silenced cells.

We observed decreased protein levels of NOB-1 in DIMT1-deficient samples (Fig. 4*E*). In contrast, lysates pulled down with an antibody to PES-1 showed robust expression levels of PES-1 in input and IP-eluted samples. Analysis of DIMT1, NOB1, and PES1 in whole-cell lysate by Western blotting was performed to confirm that NOB1 and PES1 were not affected individually in the absence of DIMT1 but their interactions were inhibited in DIMT1-deficient cells (Fig. 4*F*). This suggests that DIMT1 is playing a role in ribosomal protein interaction because NOB-1 is an integral part of the well-defined late maturation 40S ribosomal complex containing the 20S precursor to the 18S rRNA.

To extend our findings, we performed a PLA in EndoC-βH1 cells. We probed the control and *DIMT1*-silenced cells with Duolink florescent PLA probes and used two different primary antibodies to bind NOB-1 and PES-1. An increased number of PLA probes were observed in scramble siRNA control cells as compared with *DIMT1*-deficient EndoC-βH1 cells (Fig. 4*G*); these were mainly observed in the cytoplasm, where the signal from each probe reflects the extent of interactions. We did not detect any signal from the nucleus, visualized by DAPI staining, reflecting that the ribosomal maturation process is largely cytoplasmic. The levels of probes in *DIMT1*-silenced cells were 45% of those observed in the scramble siRNA-treated cells. This indicated that the two proteins are associated in the presence of DIMT1, whereas their interactions are diminished in DIMT1-deficient cells.

This circumstance may interfere with protein translation. Our observations suggest that DIMT1 deficiency impacts rRNA processing. In fact, reduced ribosome protein interaction could possibly lead to destabilization of the 40S subunit, which may fail to interact with the 60S subunit, leading to reduced mRNA translation. The latter notion is supported by our finding of reduced protein synthesis in the puromycin-based assay as well as reduced insulin content.

### DIMT1 deficiency and mitochondrial function in β-cells

**Figure 5:**
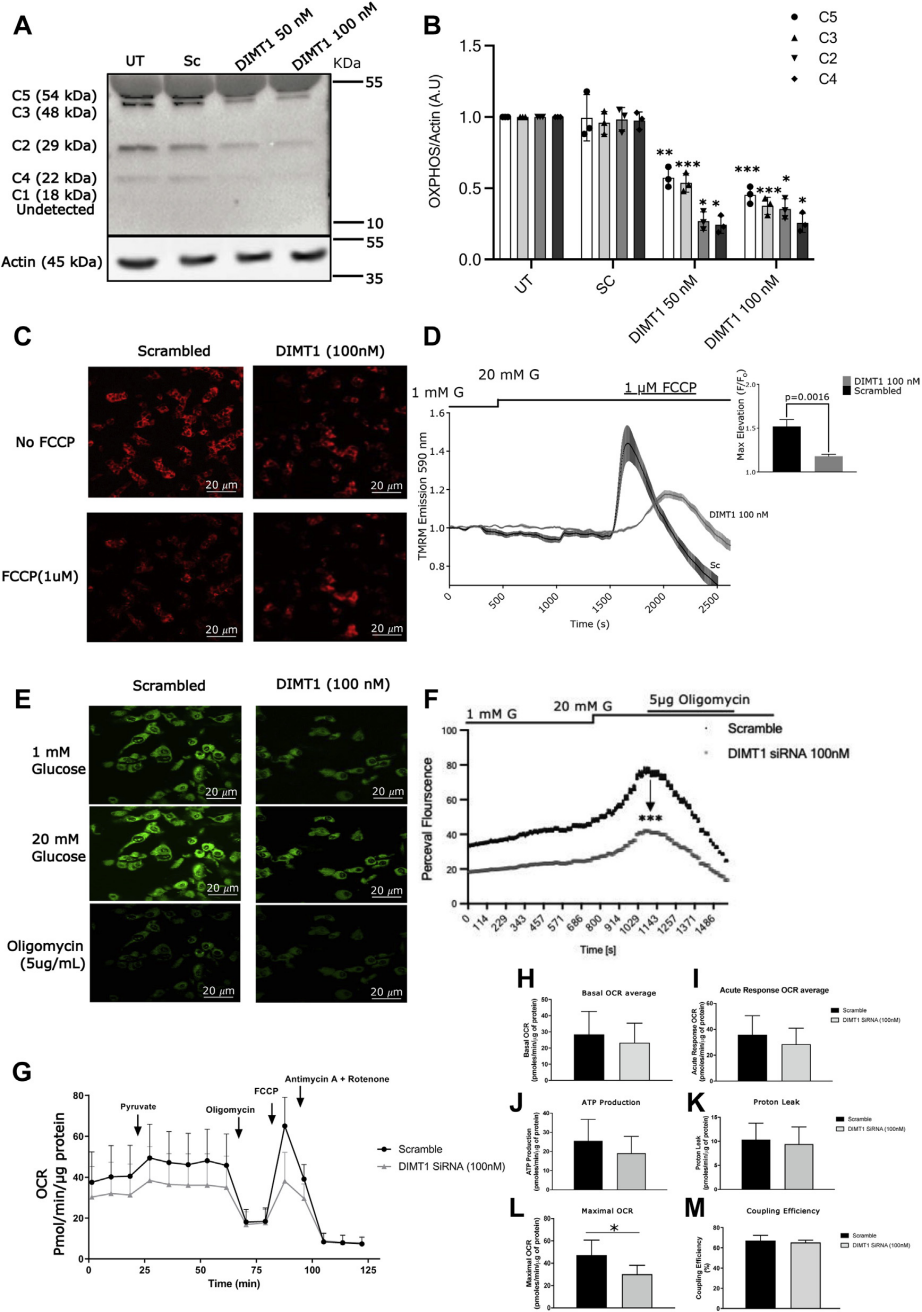
**DIMT1 and mitochondrial dysfunction in EndoC-βH1 cells.** Representative immunoblots (*A*) and densitometric analyses (*B*) of mitochondrial complex protein I–V. Data are mean ± SD (n = 3). Glucose-induced (1 and 20 mM glucose) hyperpolarization of the inner mitochondrial membrane as shown by TMRM (*C*); average traces (*thick lines*) in cells treated with scramble siRNA compared to the difference in maximal hyperpolarization with FCCP in DIMT1-silenced cells (*thin lines*) are shown (scale bars: 20 μm) (*D*; data are mean ± SD; n = 3). *E*, cytosolic ATP/ADP ratios determined by Perceval HR (*green fluorescence*); average traces and the difference in the maximal rise in ATP/ADP ratio with oligomycin, (scale bars: 20 μm). (*F*; data are mean ± SD; n = 3). *G*, mitochondrial OCR is shown upon stimulation with 10 mM pyruvate as scramble cells (*black lines*) and as DIMT1-silenced cells (*gray lines*). The pyruvate-stimulated respiratory response, proton leak, ATP production and maximal mitochondrial respiration with FCCP and nonmitochondrial respiration (antimycin+rotenone) are each expressed as fold relative to basal (*H*–*M*; data are mean ± SD; n = 4). Statistical differences were compared by Student's *t* test and extreme studentized deviate test was used to determine outliers. ∗*p* < 0.03 or <0.05 (as indicated), ∗∗*p* < 0.01 and ∗∗∗*p* < 0.001. DIMT1, dimethyladenosine transferase 1; TMRM, tetramethyl-rhodamine methyl ester.

Given that *DIMT1* knockdown perturbed GSIS in EndoC-βH1 cells, it is possible that DIMT1 deficiency disrupts mitochondrial control of stimulus–secretion coupling in β-cells. To elucidate this, we first determined the levels of the mitochondrial oxidative phosphorylation complexes (OXPHOS), using a panel of selected antibodies to both mitochondrial and nuclear-encoded proteins. We found decreased levels of OXPHOS protein expression in *DIMT1*-silenced EndoC-βH1 cells (Fig. 5, *A* and *B*). Levels of two subunits of respiratory complexes, complex V (ATP synthase) and the complex III mitochondrial-encoded (Cyt b), were markedly reduced in *DIMT1*-silenced cells (Fig. 5*A*). In addition, DIMT1 deficiency also led to a significant decrease in protein levels of the nuclear-encoded subunits of complex II (succinate dehydrogenase) and complex IV (COX1). These results suggest that DIMT1 may play a role in biosynthesis of mitochondrial OXPHOS proteins. The data also suggest that mitochondrial ribosomal biogenesis was impacted by DIMT1 deficiency in EndoC-βH1 cells.

Respiratory proteins control the electron transport chain, which extrudes protons over the inner mitochondrial membrane. Therefore, we examined the effect of *DIMT1* knockdown on the inner mitochondrial membrane potential (ΔΨm), using a fluorescent dye (tetramethyl-rhodamine methyl ester; TMRM), which is sequestered by active mitochondria and reflects the ΔΨm. Upon stimulation with 20 mM glucose, a lowering of TMRM fluorescence intensity in scrambled siRNA-treated control cells indicated a glucose-induced hyperpolarization of the inner mitochondrial membrane (Fig. 5, *C* and *D*). In contrast, *DIMT1*-deficient cells failed to produce this hyperpolarization of the inner mitochondrial membrane. In cells treated with the mitochondrial uncoupler FCCP, the increase in TMRM fluorescence intensity from baseline was significantly more pronounced in scrambled siRNA-treated control cells than in *DIMT1*-silenced cells (Fig. 5, *C* and *D*). These observations indicate that DIMT1 deficiency dissipated the ΔΨm in EndoC-βH1 cells.

As *DIMT1* knockdown led to decreased expression of ATP synthase (Fig. 5*A*), as well as reduced ΔΨm, we next asked whether *DIMT1* knockdown influences the level of mitochondrial ATP production in response to glucose. Indeed, using the ATP:ADP sensor Perceval HR, we observed a blunted increase in the ATP/ADP ratio in DIMT1-deficient cells (Fig. 5, *E* and *F*). The addition of 5 μg/ml oligomycin, an ATP synthase blocker, reduced the ATP/ADP ratio regardless of treatment. Changes in pH may affect the Perceval HR signal; therefore, data were normalized to pHRed traces. Our data suggest that disruption of DIMT1 in β-cells led to altered expression of OXPHOS complexes resulting in impaired mitochondrial ATP production.

We then analyzed the impact of DIMT1 deficiency on cellular respiration, which reflects overall mitochondrial function. We measured the oxygen consumption rate (OCR); to this end, we chose pyruvate as a substrate for mitochondrial metabolism to circumvent glycolysis. Cells treated with scramble siRNA for 48 h (standardized for optimal results as opposed to 72 h in our other experiments) raised the OCR in response to 10 mM pyruvate (Fig. 5*G*); respiratory complex inhibitors (oligomycin and antimycin + rotenone) reduced the OCR, whereas the mitochondrial membrane uncoupler FCCP increased it; this response indicated tight metabolic control of ATP synthesis on OCR (highly coupled), and high maximal mitochondrial capacity, respectively (Fig. 5, *H*–*M*). While the DIMT1-deficient cells showed a similar pattern of OCR in response to pyruvate, the maximal OCR was significantly reduced as compared with control cells (Fig. 5*G*; *p* = 0.03; n = 3). This result is in line with the deficiency of OXPHOS proteins upon *DIMT1* silencing, limiting the maximal respiratory capacity, and a reduced ability to depolarize the inner mitochondrial membrane in response to FCCP.

We also assessed the mtDNA level in DIMT1-deficient cells, but we found no change in two critical mitochondrial mRNAs, NADH-ubiquinone oxidoreductase chain 1 (*ND1*) and cytochrome c oxidase 1 (*COX1*), reflecting overall mtDNA levels. This implies that DIMT1 does not control mtDNA levels (Fig. S1*D*). Collectively, our findings suggest that *DIMT1*-silenced cells exhibit perturbed mitochondrial function that results in impaired mitochondrial metabolism and reduced insulin secretion in EndoC-βH1 cells.

### DIMT1 targets in β-cells examined by RNA sequencing

**Figure 6:**
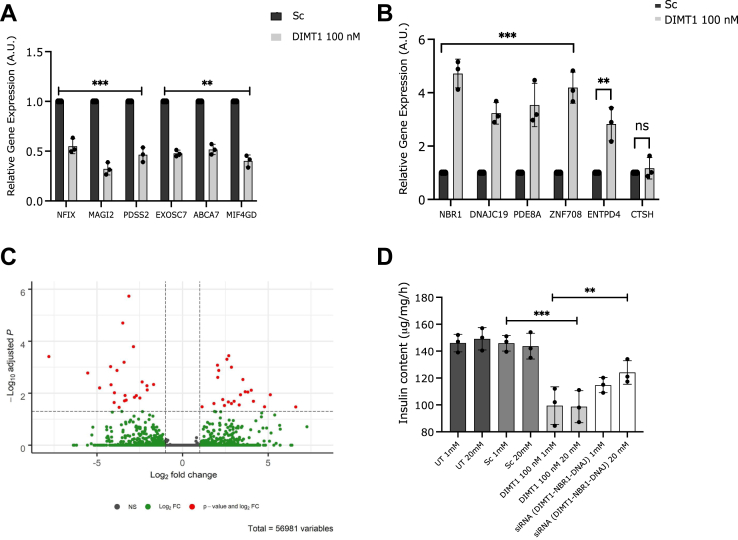
**Validation of RNA sequencing by quantitative real-time PCR.** Differentially expressed genes (DEGs) identified by RNA sequencing were evaluated; a total of six DEGs were selected based on their (i) fold change, (ii) relevance to cellular pathways, and (iii) expression levels (*A* and *B*; data are mean ± SD; n = 3). *C*, volcano plot representing significantly downregulated genes (log2fold change < (−1) and adjusted *p*-value < 0.05), which are indicated in *red* on the *left side* of the plot, while significantly upregulated genes (log2fold change > (1) and adjusted *p*-value < 0.05) are indicated in *red* on the *right side* of the plot. *Vertical dashed lines* correspond to the log2 fold change threshold of |1| and *horizontal dashed line* corresponds to the adjusted *p*-value threshold of 0.05 represented as log10 adjusted *p*-value. *D*, changes in insulin content in *NBR1*-and *DNAJC19*-silenced cells. The graphical representation of the qRT-PCR analysis and the data are expressed as mean ± SD (n = 3). Statistical analysis was done using paired Student’s *t* test. ∗∗*p* < 0.01, ∗∗∗*p* < 0.001, NS, non-significant.

To examine the effects of *DIMT1*-deficiency on the β-cell transcriptome and to identify possible targets for this protein, we performed RNA sequencing in EndoC-βH1 cells. The sequencing revealed 48 differentially expressed genes (DEGs), out of which six genes, whose expression was upregulated, and six downregulated ones were selected for validation. Downregulated mRNA expression of all the six genes was confirmed by RT-PCR in *DIMT1*-silenced cells (Fig. 6*A*). Similarly, upregulation of five of the six selected genes identified by RNA sequencing was replicated (Fig. 6*B*). *CTSH* (cathepsin H) expression was found to be nominally upregulated, but this did not reach statistical significance. Differentially expressed genes are shown in a volcano plot (Fig. 6*C*).

The differentially expressed genes upon *DIMT1* knockdown were categorized by PANTHER analysis with respect to molecular functions and biological processes; this included catalytic activity, transcription/translation regulatory activity, binding function, biological regulation, biogenesis, and metabolic process (Fig. S3). Of note, most differentially expressed genes were involved in mitochondrial function and cellular metabolism. *DNAJC19* is a mitochondrial interacting protein and plays a key role in mitochondrial function. *NAUK1* (NUAK family SNF1-like kinase 1) is required for Ca^2+^-dependent AMPK activity (26). Other upregulated genes, such as *ZNF708* (a zinc finger protein transcription factor) and *ENTPD4* (ectonucleoside triphosphate diphosphohydrolase 4), are also involved in metabolic pathways. Interestingly, we found that two of the upregulated genes, *NBR1* (neighbor of BRCA1 gene 1) and *DNAJC19* (DnaJ (Hsp40) homolog, subfamily C, member 19), are also listed in the human Mitocarta inventory of genes (Human Mitocarta 2.0, Broad Institute), further suggesting a role of DIMT1 in mitochondrial function.

In view of these results, we reasoned that upregulation of *NBR1* and *DNAJC19* could be, at least partially, responsible for the impact of DIMT1 deficiency on insulin content and protein synthesis. Reducing expression of either gene would allow us to examine this possibility and investigate their role, *e.g.*, whether they were associated with insulin content alterations. Indeed, the pathway analysis indicated that these downregulated genes were also implicated in mitochondrial function, protein synthesis, and metabolic pathways. Bearing this in mind, we investigated two targets (*NBR1* and *DNAJC19*) that fulfilled these criteria. Since we observed that *DIMT1* knockdown reduced insulin content (Fig. 3, *C* and *D*), we hypothesized that knockdown of upregulated genes secondary to DIMT1 deficiency, as identified by RNA sequencing, could ameliorate the observed reduction in insulin content in EndoC-βH1 cells. Indeed, knockdown of either of the two upregulated genes, *NBR1* and *DNAJC19*, significantly hindered the reduction in insulin content observed upon *DIMT1* knockdown (Fig. 6*D*). Knockdown of *NBR1* and *DNAJC19* did not change the expression of *DIMT1* (Fig. S1*E*). This suggests that knockdown of *DIMT1* upregulates *NBR1* and *DNAJC19* expression and that this regulation could interfere with insulin biosynthesis.

Next, we mined our RNA sequencing data to identify other rRNAs as potential targets of DIMT1. RiboMethSeq analysis showed that methylation of 28S and 5S rRNA was also impaired upon DIMT1 deficiency (Table 1), suggesting that these rRNAs could be targets of DIMT1 in β-cells. All methylated sites and differentially altered genes revealed by the RiboMethSeq of scrambled siRNA-treated and *DIMT1* knockdown cells are found in Tables S2 and S3. The functional implications of methylation of 28S and 5S rRNA by *DIMT1* in β-cells remain to be explored.

**Table-1:** Methylated sites present in control samples but not in samples after DIMT1 knockdown

Gene	Methylated position	Strand	Methylated site	Riboscore
RNA5S9	92	_	Cm	0.8058252
RNA5S9	4	_	Um	0.7700288
RNA28SN2	2397	+	Um	0.7839793
RNA28SN2	2403	+	Um	0.9242122
RNA28SN2	2420	+	Um	0.8673416
RNA28SN2	2465	+	Am	0.7885364
RNA28SN4	3333	+	Cm	0.8511263
RNA28SN4	4978	+	Um	0.8689244
RNA5S9	35	-	Um	0.7962092

The RiboMethSeq protocol in (ebook ISBN 978-1-4939-6807-7) was applied to generate transcript reads, which were aligned to the human rRNA DNA sequences (5S, 5.8S, 18S, 28S) found in the NCBI reference human genome (GRCh38) by use of Bowtie 2 (PMID: 22388286).

The R package RNAmodR.RiboMethSeq (v. 1.2.0) was used for counting of both 5′ and 3′ ends of mapped reads and calculating the Score Mean and RiboMeth-seq Score (Riboscore) as defined in (PMID: 25417815, PMID: 30539563). A site with a minimum Score Mean of 0.60 and a minimum RiboMeth-seq score of 0.75 was considered methylated.

To validate our RNA sequencing, we also performed a gene expression analysis of key β-cell expression signatures. Out of four genes tested (pancreatic and duodenal homeobox 1 (*PDX1*), lactate dehydrogenase A (*LDHA*), MAF BZIP transcription factor A (*MAFA*), and insulin (*INS1*)), we could only detect a downregulation of *INS-1* mRNA in DIMT1-deficient cells (Fig. S1*F*). Downregulation of *INS1* transcripts could be an additional target of DIMT1 further impacting insulin secretion.

## Discussion

rRNA modification is highly conserved across species; only a handful of organisms are known to lack it (27, 28). The specific location of the modified residues on the rRNA within the ribosome and its conservation across the species are of great importance and likely play a critical role in translational regulation and rRNA processing. This primarily occurs in the nucleolus, but a part of the late maturation also occurs in cytoplasm, requiring an ensemble of associated proteins that facilitate ribosomal assembly and transport of the preribosomal complex from the nucleolus to the cytoplasm (29, 30). Intricate tethering ensures the association of rRNAs to ribosomal proteins (r-proteins), which is regulated by the coordination of substrates and additional factors (31). This process is mainly governed by two discrete ribosomal subunits, *i.e.*, the 60S and 40S rRNA that associate during translation initiation to form the functional ribosome. The ribosomal subunits and the other components of the translational machinery are typically modified by methyltransferases. The methylations mainly occur on rRNA, transfer RNA (tRNA), messenger RNA (mRNA), translation factors, and r-proteins. Methylation of these RNA components of the ribosomes is crucial for subunit stabilization, structure stability, translational fidelity, ribosome synthesis and, consequently, for cellular protein synthesis (32, 33).

Here, we report that N^6-^N^6^ dimethylation of two adenosine residues on 18S rRNA by DIMT1 is part of the control of β-cell translation. Our data revealed a dependence of protein synthesis on methylation executed by DIMT1. DIMT1 is highly conserved among species, which suggests that it likely plays a specific functional role in cells in general, including pancreatic β-cells. We have therefore assessed the role of DIMT1 in β-cells and demonstrated that methylation of rRNA by DIMT1 is involved in rRNA processing. Two important proteins associated with the 60S and 40S rRNA (NOB1 and PES1) were found to be altered in DIMT1-deficient cells. Of note, pre-40S ribosomal biogenesis in humans requires cleavage by NOB1 at the 3′end of 18S rRNA (34). This step ensures the production of properly assembled 40S rRNA subunits. We found that DIMT1 is involved in β-cell protein translation: loss of DIMT1 significantly attenuated protein synthesis. The mechanism by which DIMT1 impacted protein synthesis is still not fully resolved, but our study suggested that loss of DIMT1 leads to immature rRNA formation—mainly 28S, 18S, and 5.8S—that may perturb rRNA processing, in part, through reduced interaction of NOB-1 and PES-1 of 40 and 60S ribosomal subunits. It has previously been suggested that regulation of NOB-1 in yeast mediates cleavage of site D in the pre-18S rRNA, which is mediated by internal transcribed spacer 1; this facilitates NOB-1 access to its cleavage site (35, 36, 37). Dim2, the yeast homolog of DIMT1, has also been shown to interact with Nob-1 (38), assisting in rRNA processing. Defects in pre-RNA processing could lead to cell cycle arrest and apoptosis followed by ribosomopathies (39). Interestingly, our data from rat islets and human β-cells suggest that regulation of ribosomal biogenesis by rRNA maturation, and NOB-1 and PES-1 interaction, is mediated by DIMT1. High-throughput experimental strategies, such as mass spectrometric analyses (*e.g.*, SILAC), and polyribosome profiling could provide additional information about how these modifications regulate protein synthesis.

TFB1M, a nuclear-encoded protein and a DIMT1 homolog, has previously been reported to control protein translation in β-cell mitochondria (18); its mRNA and protein expression is downregulated in islets from T2D donors. This notion further prompted us to examine the expression of *DIMT1* in human islets and characterize its functional implications in β-cells. In contrast to *TFB1M*, expression of *DIMT1* was increased in islets from T2D donors and correlated positively with HbA1c. Similarly, *DIMT1* expression correlated negatively with the secretion of insulin but positively with insulin mRNA expression. While these observations imply that expression of *DIMT1* reflects glycemia, some apparent contradictions have not been resolved by our studies. Here, we showed that the knockdown of DIMT1 in rodent and human β-cell lines, as well as in rat islets, results in impaired insulin secretion and content; given the correlations with these traits in human islets, there is an agreement with the impact on content but not secretion of insulin. Clearly, the experimental situation in cells differs from the chronic situation in islets donated by deceased T2D patients, which also are heterogeneous with regard to cellular composition; rRNAs methylated in rodent and human cells and in islets may not be the same. This notwithstanding, expression of both these dimethylases, TFB1M and DIMT1, is regulated in T2D islets.

In contrast to TFB1M, which dimethylates mitochondrial 12S rRNA (17), DIMT1 methylates 18S rRNA in the cytoplasm (25). This notwithstanding, and given its homology to TFB1M, we examined whether effects on cytosolic rRNA impacted mitochondrial function. To this end, we chose four parameters of mitochondrial function (OXPHOS, mtATP, ΔΨm, and OCR) and assessed whether they were affected by DIMT1-deficiency. We found that deficiency of DIMT1 in β-cells reduced the levels of mitochondrial-encoded proteins, which most likely is due to an abrogation of protein synthesis. This effect could be explained by the observed reduction in adenosine dimethylation of the 18S rRNA of the 40S subunit in the cytoplasm in *DIMT1*-silenced cells, which is expected to result in ribosomal biogenesis defects. In addition, there was a significant impact of DIMT1-deficiency on both mitochondrial and nuclear-encoded mitochondrial complex subunits. Our data suggest that defects in cytosolic 18S rRNA lead to a perturbation of mitochondrial complexes and a nuclear response. Together, the cytosolic and mitochondrial impact may result in a general decrease in mitochondrial function. It has also been shown that mitochondrial-encoded proteins stabilize some nuclear-encoded proteins (40). It is of interest to investigate the interdependency of these two axes.

In view of these findings, we examined whether defects in mitochondrial complexes translate into downstream effects. *DIMT1* knockdown perturbed complex V (ATP Synthase); we found impaired mitochondrial function illustrated by dissipated ΔΨm, reduced levels of mtATP, and reduced OCR. These impairments in mitochondrial function are most likely due to reduced electron transport and subsequently OXPHOS, leading to mitochondrial dysfunction in DIMT1-deficient EndoC-βH1 cells. As a result, β-cell stimulus–secretion coupling was perturbed followed by a reduction in insulin release and content. Our findings identify β-cell translation as a regulatory process, generating proteins required for cellular and mitochondrial function. When impacted, as observed upon DIMT1 deficiency, impaired protein synthesis, mitochondrial dysfunction, and impaired insulin secretion may evolve. Our results are in line with our previous observations on the role of TFB1M in β-cells and its involvement in the pathogenesis of T2D (18).

We further characterized the functional relevance of DIMT1 methylation as one component of β-cell translation. We showed that DIMT1 deficiency leads to a defect in the ribosomal proteins NOB1 and PES1 that may lead to attenuation of β-cell protein synthesis. This finding demonstrated the conservation of DIMT1 function in human β-cells, similar to that found in yeast cells exerted by Dim1. In fact, Dim1, the yeast counterpart of human DIMT1, also mediates dimethylation at the 3′-end for preribosomal RNA processing (41). Dimethylation in other cells, such as HeLa, has previously been reported as a critical step in late 18S rRNA modification (42); its functional relevance in protein synthesis was not studied. Using primer extension, amplifying a sequence flanking the region of DIMT1 methylation on 18S rRNA, we found that dimethylation occurs also in β-cells. We also found lower levels of 28S, 18S, and 5.8S rRNA in DIMT1-deficient islets cells that may underlie defective rRNA maturation.

**Figure 7:**
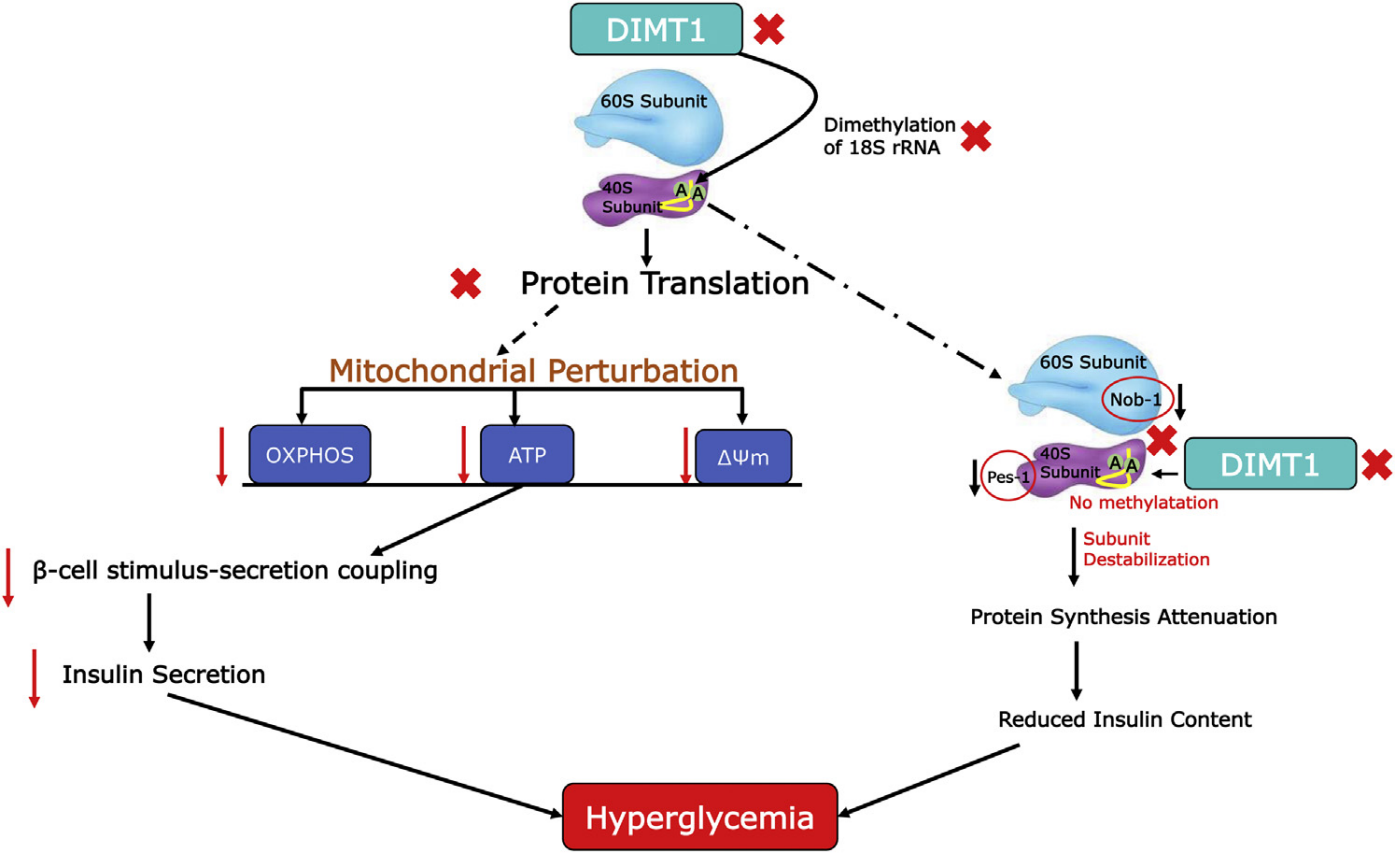
**Schematic diagram of DIMT1-mediated β-cell dysfunction.** Deficiency of DIMT1 may lead to attenuation of cellular protein synthesis and mitochondrial dysfunction, which both contribute to the pathogenesis of T2D. 18S rRNA methylation by DIMT1 is instrumental in rRNA processing and protein translation. In the event this is perturbed, translation of proteins required for mitochondrial functions, such as OXPHOS, ATP production and ΔΨm, is impacted. Consequently, mitochondrial dysfunction causes defects in insulin secretion that lead to hyperglycemia. In parallel, deficiency of DIMT1 leads to rRNA processing defects and also to an interference of the interaction between NOB-1 and PES-1. This interaction is crucial for late rRNA maturation and efficient translation, which, when hindered, may lead to further perturbation in overall protein synthesis. Attenuation of cellular protein synthesis is likely to impact insulin content, which contributes to the hyperglycemic condition. ΔΨm, mitochondrial membrane potential; ATP, adenosine triphosphate; NOB-1, NIN1 (RPN12) binding protein 1 Homolog; OXPHOS, mitochondrial oxidative phosphorylation; PES-1, pescadillo ribosomal biogenesis factor 1; rRNA, ribosomal RNA.

Several ribosomal assembly factors have been discovered in yeast and human cells (39, 43). The exact role of the 18S rRNA modification in β-cell ribosome function was not completely resolved. To fully understand the complex pathways of ribosome assembly will require the assessment of the precise function of these methyltransferases, identification of substrates, their binding sites, and their functional relevance in cellular pathways. High-throughput protein–protein interaction methods, ribosome profiling, and mass-spectrometry-based analyses will be helpful to further elucidate β-cell translation. One limitation of our study is the use of only one siRNA out of three tested for the functional experiments upon knockdown. However, given the consistency of the results, generating our current working model (Fig. 7), off target effects of our *DIMT1* siRNA are unlikely (as supported by the use of another set of siRNA SMARTpool for one of the functional experiment Fig. S1*C*) as well as by BLAST search for other relevant target sequences of the *DIMT1* siRNA2) to account for the functions of DIMT1 in β-cells reported here. In sum, our work has identified a role of a highly conserved rRNA methyltransferase in human β-cell translation with subsequent functional implications.

## Experimental procedures

### Cell lines, islets, and reagents

EndoC-βH1 cells (Endo Cells; used by permission of Endo Cell) were grown on Matrigel-fibronectin coated (100 μg/ml and 2 μg/ml, respectively, Sigma-Aldrich) cell culture plates in DMEM containing 5.6 mM glucose, 2% BSA fraction V (Roche Diagnostics), 10 mM nicotinamide (Merck Millipore), 50 μM 2-mercaptoethanol, 5.5 μg/ml transferrin, 6.7 ng/ml sodium selenite (Sigma-Aldrich), 100 U/ml penicillin, and 100 μg/ml streptomycin (PAA Laboratories). INS-1832/13 cells were cultured as described (44). Rat pancreatic islets were isolated and hand-picked as previously described (45). Islets were cultured in RPMI 1640 with GlutaMax (Gibco-BRL, DK), supplemented with 10% FCS (Gibco-BRL, DK), 100 U/ml penicillin, 100 μg/ml streptomycin, and 5 mM glucose. Islets were transfected by Lipofectamine RNAimax and 100 nM rat *DITM1* siRNA for 24 h. The day after the first transfection, the islets were transferred into new plates and retransfected for another 24 h. GSIS and total insulin content were determined 72 h after the first transfection. The protein estimation kit used was from Pierce, Thermo Scientific. All primary antibodies were from Abcam, and the appropriate secondary antibodies used were from Santa Cruz (Santa Cruz Biotechnology, Inc.). All reagents used for qRT-PCR were from Thermo Scientific. The TaqMan quantitative real-time PCR (qRT-PCR) master mix was from Applied Biosystems. The control (scramble) and human *DIMT1* siRNA (Cat# 4392420 and 4392421) were from Thermo Scientific, and Rat *DIMT1* siRNA (Cat# NM_001106408 (SASI_Rn02_00218668) was from (Sigma Aldrich). SMARTpool siRNA (Cat# 294718) were procured from (Dharmacon). Fluorescent probes were supplied by Molecular Probes (Rockford, IL). Insulin was measured by ELISA (Mercodia). All other chemicals were from Sigma Aldrich.

RNA sequencing of human islets was performed on 155 nondiabetic and 33 T2D islet donors (20). Human islets were acquired from a collaboration with the Nordic Network of Clinical Islet Transplantation. Complete datasets are available at public accessible repository (EGA; https://www.ebi.ac.uk/ega) with accession numbers: EGAS00001004042. Data were extracted from the Human tissue laboratory, Lund University Diabetes Centre, approved by Malmö/Lund Ethics Committee on Animal Testing at the Lund District Court, with permission to utilize data in accordance with in-house guidelines (20). Comparisons and correlations between diabetic and nondiabetic islet donors were performed as previously described (20). Briefly, raw expression sequence files were aligned to the human genome hg38, using STAR 2.4.1; gene-level counts were obtained using feature counts from the subread package. Counts were normalized to account for sequencing depth, log-transformed, and differential expression analysis was performed in edgeR. For gene–gene correlations, counts adjusted for gene-length (FPKM values) were used and normalized to account for sequencing depth and distribution. Linear models were applied to calculate relationships between variables and outcomes as described previously (20).

### Cell incubations

EndoC βH1-cells were grown in DMEM culture medium with 5.6 mM glucose. Islets and INS-1 832/13 cells were cultured in RPMI medium with 5 and 11.1 mM glucose, respectively. We used silence select negative control No. 1 siRNA (Thermo Scientific; Cat# 4390844) with a concentration of 10 nM as suggested by the manufacturer. Both cell lines were incubated in the absence and presence of *DIMT1* siRNA at 0, 50, and 100 nM concentrations for 48 or 72 h; whereas islets were transfected with 100 nM of *DIMT1* siRNA for 72 h by Lipofectamine RNAi Max (Invitrogen) according to the manufacturer’s instructions. We used predesigned *DIMT1* siRNA (Thermo Scientific) Cat# 4392420 and 4392421 for EndoC-βH1 cells (Sense: GGAUGGUCUAGUAAGGAUAtt), (Antisense: UAUCCUUACUAGACCAUCCca), and Cat# SASI_Rn02_00218668 (Sigma Aldrich) for islets and INS-1832/13 cells (Sense: CUGUUCAGUACAGAAUACUdt), (Antisense: AGUAUUCUGUACUGAACAGdt). For *NBR1*, we used Cat# SASI_Hs01_00169473, (Sense: GCUUAAGAUGGCAGUUAAA[dT][dT]) and (Antisense: UUUAACUGCCAUCUUAAGC[dT][dT]) and for *DNAJC19* Cat# SASI_Hs01_00055864, (Sense:CAGCAUUAAUACUAGGUGU[dT][dT]) and (Antisense: ACACCUAGUAUUAAUGCUG[dT][dT]) (Sigma Aldrich). For rRNA we used primers from Eurofins, (Sense: AGAGGTAAACGGGTGGGGTC) and (Antisense: GGGGTCGGGAGGAACGG) for 28S, (Sense:AAACGGCTACCACATCCAAG) and (Antisense: TACAGGGCCTCGAAAGAGTC) for 18S, (Sense:ACTCGGCTCGTGCGTC) and (Antisense: GCGACGCTCAGACAG) for 5.8S, and (Sense: CCCCTGCTAGAACCTCCAAA) and (Antisense: TGTCTGGAGTCTTGGAAGCT) for 7SK. On termination of incubation, cells were assessed for cell viability with trypan blue; other assays were performed as described below.

### RNA isolation, RNA electrophoresis, and quantitative real-time PCR

Total RNA was extracted from the cells, using TRI Reagent (Sigma Aldrich) according to the manufacturer’s instructions. RNA concentrations were determined by NanoDrop Spectrophotometer (Thermo Scientific). Equal quantities of total RNA were reversely transcribed using the RevertAid First-Strand cDNA synthesis kit (Fermentas) in reactions containing 500 ng of total RNA. qRT-PCR was performed using TaqMan gene expression assays (Human-Assay ID Hs00917510_m1; and (Rat-Assay ID Rn01489483_m1; Applied Biosystems, Life Technologies). ND1 Taqman gene expression (Human-Assay ID Hs02596873_s1; and COX-1 Human Assay ID Hs02596864_g1 were procured from Applied Biosystems, Life Technologies). PDX-1, LDHA, MAFA, and INS-1 were also procured from (Applied Biosystems, Life Technologies). For rRNA gene expression, SYBRgreen mastermix was used from (BioRad). Gene expression was quantified by the comparative Ct value, in which the amount of target is expressed as 2^−ΔΔCt^ using 7SK as a reference gene. For analysis of 28S, 18S rRNA, 2 μg of total RNA were resolved on denaturing agarose gels (6% formaldehyde, 1% agarose in 1x MOPS buffer). Gels were run for 2 h at 80 V. For the loading control (tRNA), the same amount was loaded on denaturing acrylamide gels (6% TBE-Urea gel run for 1 h at 180 V in 1x TBE buffer).

### Western blot analysis

Cells were washed with phosphate-buffered saline (PBS) and homogenized in cell lysis buffer (150 mM NaCl, 1% NP40, 10% DOC, 10% SDS, 50 mM Tris, pH 7.4). Determination of protein concentration was performed using the BCA protein assay kit (Pierce). Samples were mixed with standard Laemmli loading buffer and 30 μg protein loaded on mini-Protean TGX precast gel (Bio-Rad). After SDS-PAGE, proteins were transferred to Immunoblot PVDF membranes (BioRad), the membrane blocked with 3% BSA, and further incubated with rabbit monoclonal anti-DIMT1 antibody (1:1000 dilution) and Rabbit polyclonal anti-OXPHOS (1:250 dilution antibody; both from Abcam), anti-NBR1 and anti-DNAJC19 (1:2000 dilution antibody; both from Sigma Aldrich), anti-NOB1 and anti-PES1 (1:1000 dilution; Sigma Aldrich) Tubulin and β-actin antibody (1:5000 dilution; Abcam) was used as a loading control. Horseradish-peroxidase-linked goat anti-rabbit IgG (1:5000 dilution; Santa-Cruz Biotechnology) was used as a secondary antibody. Blots were developed with enhanced chemiluminescence (ECL). Densitometry analysis was performed using BioRad ImageLab software.

### Insulin secretion and content

After 72 h of scramble and siRNA treatment, confluent EndoC-βH1 cells were starved in medium at 2.8 mM glucose overnight and incubated next day in 1X secretion assay buffer (SAB) (114 mM NaCl, 1.2 mM KH_2_PO_4_, 4.7 mM KCl, 1.16 mM MgSO_4_, 2.5 mM CaCl2, 100 mM HEPES,25 mM NaHCO_3_ 0.2% BSA, pH 7.4) containing 1 mM glucose for 2 h. Finally, cells were incubated in SAB containing 1 and 20 mM glucose concentrations for 1 h. For islets and INS-1832/13 cells, we used a similar SAB buffer and 2.8 mM as basal and 16.7 mM as stimulatory glucose concentration. After the incubation, supernatants and lysates were collected. Insulin secretion and content were measured by Human (#10-1113-01) and Rat Insulin ELISA Kit (#10-1250-01) (Mercodia) according to the manufacturer’s instructions.

### Protein synthesis measurement

EndoC-βH1 cells were seeded in a 96 well dish with a density of 5 × 10^4^ cells and transfected with *DIMT1* siRNA. At confluence, cells were harvested by centrifugation (400*g*, 5 min), and the supernatant was carefully removed; cells were resuspended in 500 μl medium containing 25 μM OPP (#601100, Cayman Chemicals). Subsequently, cells were incubated for 30 min at 37  °C and 5% CO_2_. After incubation, cells were centrifuged at (400*g*, 5 min), and supernatants were removed. One-hundred microliters of cell-based fixatives was added to each well and incubated for 5 min at room temperature. Cells were then centrifuged and 100 μl of wash buffer was added, and cells were incubated for another 5 min and centrifuged again. One-hundred microliters of five FAM-Azide solutions were added and cells were incubated for 30 min. After the final wash, cells were examined in a fluorescent plate reader using a filter designed to detect FITC (excitation/emission= 485/535 nm). Three specificity controls were included: (1) cells labeled with OPP; (2) knockdown cells labeled with OPP and; 3) cells incubated with cycloheximide at a final concentration of 50 μg/ml for 15 min prior to, as well as during incubation with OPP.

### Primer extension assay to assess methylation

For the primer extension assay, sense and antisense primers were designed flanking the methylated adenosine residues on human 18s rRNA. Hemo KlenTaq polymerase (Cat# M0332S, NEB) was used, which is a mutant DNA polymerase with reverse transcriptase activity and whose processivity is blocked by the presence of RNA methylation. Besides, we chose reverse transcriptase M-MuLV as a positive control that is not affected by the modification and amplifies the same RNA. qRT-PCRs were performed in a total volume of 20 μl reaction mixture containing 100 nM of Taqman gene expression assay (Human-Assay ID Hs00917510_m1), 200 μM dNTPs (each), in KlenTaq reaction buffer. The concentrations of the different RNAs were quantified, and 500 ng of each sample was reverse transcribed as explained before. The primer sets used for the detection of the methylated adenosine residues were: 1) *DIMT1* Forward *GACGGTCGAACTTGACTATCTA*; 2) *DIMT1* Reverse *AATGATCCTTCCGCAGGT*, and 3) probe *AGTCGTAACAAGGTTTCCGTAGGTGA*. Gene expression was quantified by the comparative Ct values, in which the amount of target is expressed as 2^−ΔΔCt^ using actin as a reference gene.

### Mitochondrial membrane potential (ΔΨm) measurement

EndoC-βH1 cells were seeded onto collagen-coated 8-well chambered cover glasses (Lab-Tek, Thermo Scientific) at a density of 70,000 cells/cm^2^. After 24 h, cells were transfected with 100 nM of *DIMT1* siRNA and incubated further for 72 h. For the ΔΨm measurement, cells were preincubated with imaging buffer (135 mM NaCl, 3.6 mM KCl, 1.5 mM CaCl_2_, 0.5 mM MgSO_4_, 0.5 mM Na_2_HPO_4_, 10 mM HEPES, 5 mM NaHCO_3_, pH 7.4) containing 1 mM glucose for 2 h with 100 nM of tetramethylrhodamine methyl ester (TMRM; Invitrogen). This imaging buffer formulation was standardized for the microscopy studies. After the incubation, cells were washed again with the imaging buffer only and subjected to live-cell confocal microscopy in quench mode, where the whole-cell fluorescence decreases upon mitochondrial hyperpolarization. After recording the basal level of TMRM fluorescence in 1 mM glucose, cells were switched to 20 mM glucose. Carbonyl cyanide-4-phenylhydrazone (FCCP) was used to dissipate the ΔΨm. Zeiss LSM510 inverted confocal fluorescence microscope with 543 nm excitation and 585 nm long pass emission settings were used to record the data, which were background corrected and normalized.

### ATP:ADP measurement

Single-cell ATP/ADP ratio measurements were carried out, using coexpression of a pericam-based ATP biosensor (Perceval HR; Addgene ID: 49083) and pHRed. EndoC-βH1 cells were seeded onto collagen-coated 8-well chambered cover glasses (Lab-Tek, Thermo Scientific) at a density of 70,000 cells/cm^2^. After 24 h, cells were cotransfected with 1 μg of plasmid encoding Perceval HR (Addgene ID:49,083) and 100 nM of *DIMT1* siRNA at 50% cell confluency. Cells were grown for 72 h before measurements; cells were preincubated at 37 °C in 400 μl of imaging buffer (135 mM NaCl, 3.6 mM KCl, 1.5 mM CaCl_2_, 0.5 mM MgSO_4_, 0.5 mM Na_2_HPO_4_, 10 mM HEPES, 5 mM NaHCO_3_, pH 7.4) containing 1 mM glucose. After recording the basal signal from Perceval HR (1 mM glucose), 20 mM glucose was added. Cells were imaged with 490 nm excitation and 535 nm emission filter settings for Perceval and pHRed was excited using 578/16 and 445/20 nm bandpass filters; the emissions were collected through a 629/56 nm bandpass filter on Zeiss LSM510 inverted confocal fluorescence microscope.

### Respiration

Mitochondrial OCR was determined in EndoC-βH1 cells (70,000 cells/cm^2^), using the Seahorse Extracellular Flux Analyzer XF24 (Seahorse Bioscience). After transfection (48 h) with *DIMT1* or scramble siRNA, the cell culture medium was exchanged for 500 μl of Seahorse assay buffer supplemented with 1 mM glucose for 2 h at 37 °C. OCR was recorded in intact cells stimulated by 10 mM pyruvate. Oligomycin (OM)-independent OCR (5 μg/ml) was measured. The mitochondrial inner membrane ionophore (FCCP, 4 μM) was added to determine maximal respiratory capacity, and rotenone+antimycin (1 μM) was added to block the transfer of electrons from complex I to ubiquinone. Data were analyzed by the Seahorse wave software (Agilent).

### Immunoprecipitation of binding partners

EndoC-βH1 cells were treated with *DIMT1* siRNA and lysates were isolated using a proprietary lysis buffer (Pierce, Thermo Scientific). Co-IP was performed at 4 °C unless otherwise indicated, using a Pierce spin column. The binding of the NOB1 antibody to protein A/G agarose beads was performed according to the manufacturer’s instructions. Protein A/G agarose slurry (20 μl) was washed twice with 200 μl PBS buffer and incubated with 100 μl NOB1 antibody prepared in PBS (10 μl NOB1 antibody +85 μl H_2_O + 5 μl 20X PBS) at 25 °C for 30 min on a gentle shaker. The supernatants were discarded, and the beads were washed three times with 300 μl PBS. After removing the supernatant, beads were washed three times again. The antibody-cross-linked beads were incubated overnight at 4 °C with 500 μl of 1 mg lysates, which were precleared with control agarose resin (Pierce) for 2 h on a shaker. After removing supernatant (flow-through) and washing with 600 μl washing buffer five times, the immunoprecipitates were eluted with 40 μl 2X-Laemmli buffer at 100 °C for 10 min. Equal amounts of eluted input and co-IP lysates complex were subjected to SDS-PAGE separation for Western blotting.

### Proximity ligation assay for protein–protein interaction

For PLA, DuoLink PLA technology probes and reagents (Cat# DUO92101-1KT) (Sigma-Aldrich) were used. Cells were permeabilized using the combination of paraformaldehyde, methanol, and ethanol incubation for 10 min. After two washes in PBS, cells were incubated with blocking solution for 30 min at 37 °C and then with the two different primary antibodies (NOB1 and PES-1) for 1 h at room temperature. The coverslips were washed twice for 5 min with buffer A, followed by incubation with the PLA probes (secondary antibodies against two different species bound to two oligonucleotides: anti-mouse MINUS and anti-rabbit PLUS) in antibody diluent for 60 min at 37 °C. After two washes of 5 min with buffer A, the ligation step was performed for 30 min at 37 °C. The cells were washed with buffer A twice for 2 min before incubation for 100 min with amplification stock solution at 37 °C. The amplification stock solution contains polymerase for the rolling circle amplification step and oligonucleotides labeled with fluorophores, which will bind to the product of the rolling circle amplification and thus allowing detection. After two washes of 10 min with buffer B, cells were incubated with FITC-conjugated buffers. Finally, the coverslips were washed with PBS and mounted with Duolink *in situ* mounting medium containing DAPI. The cells were visualized under fluorescent microscopy with UV lasers for the nucleus and TRITC for the detection of red PLA signals (excitation wavelength of 594 nm and an emission wavelength of 624 nm).

### RNA sequencing

RNA was extracted from EndoC-βH1 cells, and sequencing was performed using Illumina RiboMethSeq Protocol (ebook ISBN 978-1-4939-6807-7, Chapter 12) (46). The library was prepared by the NEB-Next multiplex small RNA library prep set for Illumina. The libraries were loaded and sequenced on Illumina NextSeq500 sequencer in the sequencing core facility at Lund University Diabetes Centre. Transcript reads were mapped to the human transcriptome (Gencode Release 30) and quantified with Salmon (v.0.14.0). DEGs were identified with DESeq2 (v.1.25.10). Detailed schematic of RNA sequencing protocol and analysis is depicted in (Fig. S4).

### Panther pathway analysis

Significantly altered genes were functionally classified *via* molecular function and biological processes, using the PANTHER classification system (http://www.pantherdb.org). The candidate gene list was converted into a text file (ID list) with *Homo sapiens* selected as the organism database; functional classification was viewed in a pie chart. PANTHER classifies genes based on published experimental evidence to predict functions. To assess our dataset relative to the global set of human genes, binomial statistics and Fischer’s exact test for multiple testing within the PANTHER system were applied. An outline of the detailed study design is depicted in (Fig. S3).

### Statistical analysis

Mean ± SD were calculated. Pairwise comparisons were made by *Chi-square*-test; to determine the statistical significance between two groups, parametric or nonparametric tests were used as indicated. A significance level of *p* < 0.05 was considered statistically significant.

## Data and resource availability

The datasets generated in the current study are available from the corresponding authors upon request.

## Supporting information

This article contains supporting information.

1. Relevant SNPs mapping to the *DIMT1* region Table S1.
2. List of the methylated sites and differentially altered genes from RNA sequencing in control and knockdown cells Table S2.
3. List of the differentially expressed genes after *DIMT1* knockdown Table S3.
4. Cell viability assay by trypan blue, mRNA, and protein expression data (Fig. S1), Primer extension method schematic (Fig. S2), List of the genes analyzed by PANTHER pathways (Fig. S3) and RNA sequencing step by step process (Fig. S4).

## Conflict of interest

The authors declare that there is no conflict of interest with the contents of this article.

## Supporting information

Supplementary Figures 1–4

Supplementary Figures and Tables Captions
