# Supplementary material for: Ribosomal biogenesis regulator DIMT1 controls β-cell protein synthesis, mitochondrial function, and insulin secretion
Source: J Biol Chem. 2022 Feb 8;298(3):101692. doi: 10.1016/j.jbc.2022.101692 (PMC8913306; doi:10.1016/j.jbc.2022.101692)

Supplementary Figure S1

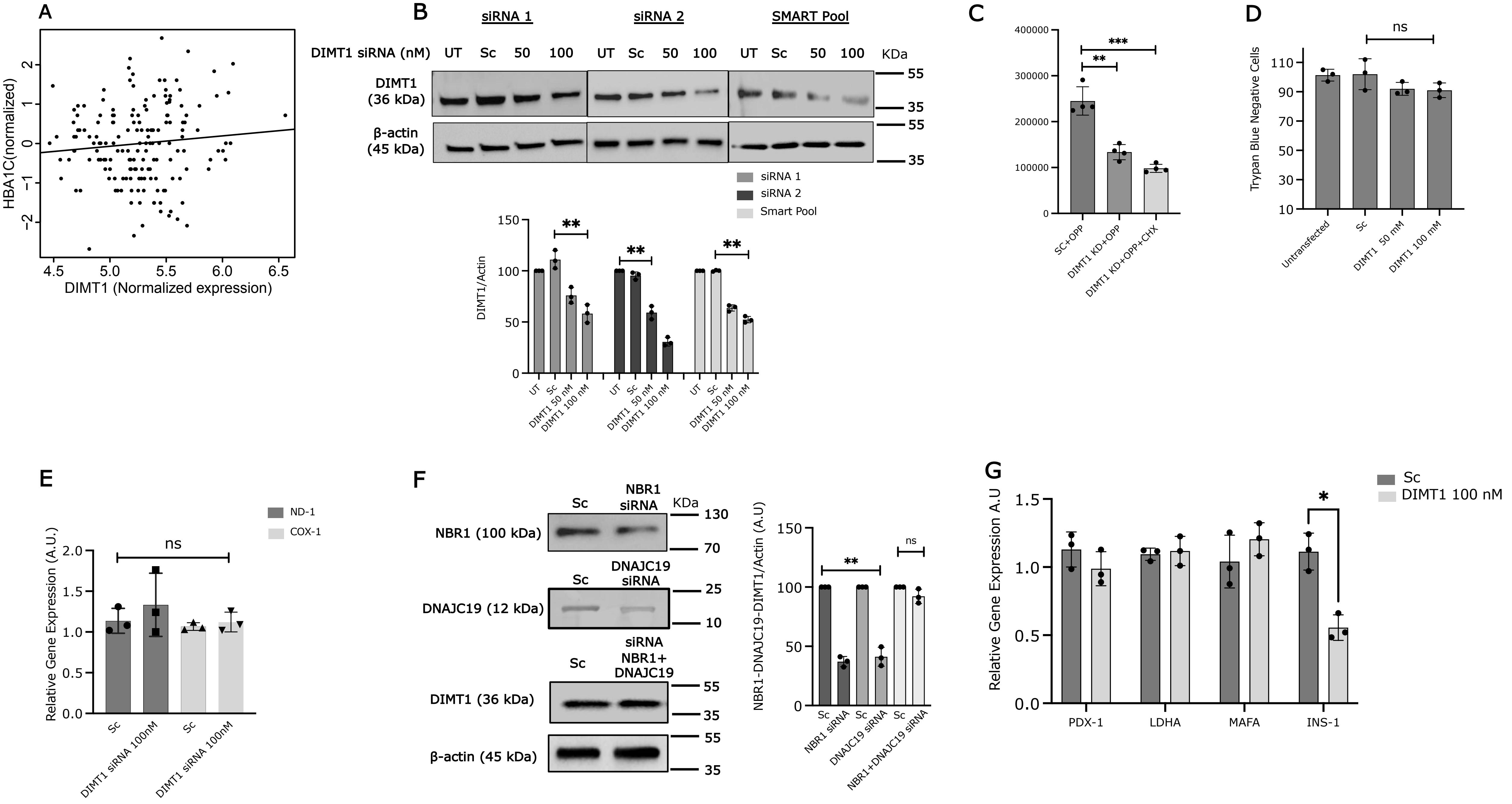

Supplementary Figure S2

Primer Extension Method Schematic.

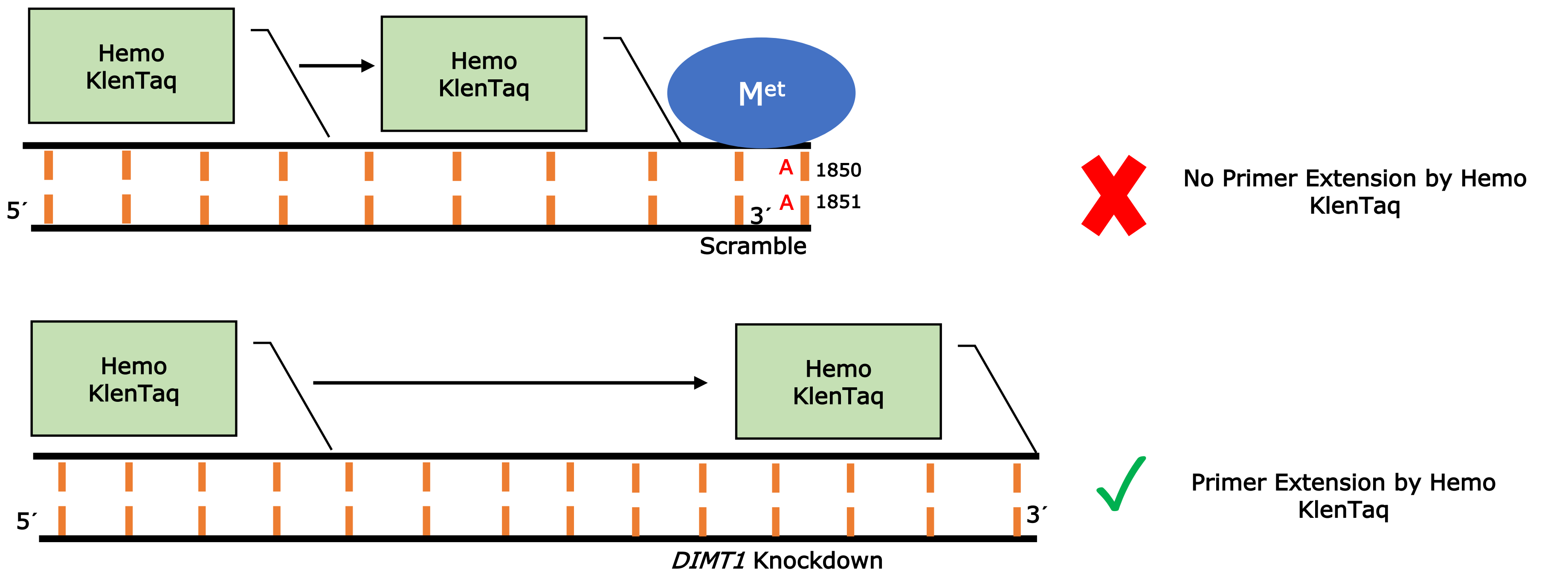

The relative gene expression ratio (*R*) of a target gene is calculated based on efficiency (E) and the cycle threshold (Ct) deviation of an unknown sample versus a control, and expressed in comparison to a reference gene;

$$\text{Ratio} = \frac{E_{(\text{Target})}^{\Delta Ct\ \text{Target(Control-Target)}}}{E_{(\text{Ref})}^{\Delta Ct\ \text{Ref (Control-Target)}}}$$

Supplementary Figure S3

PANTHER Pathway Analysis.

A

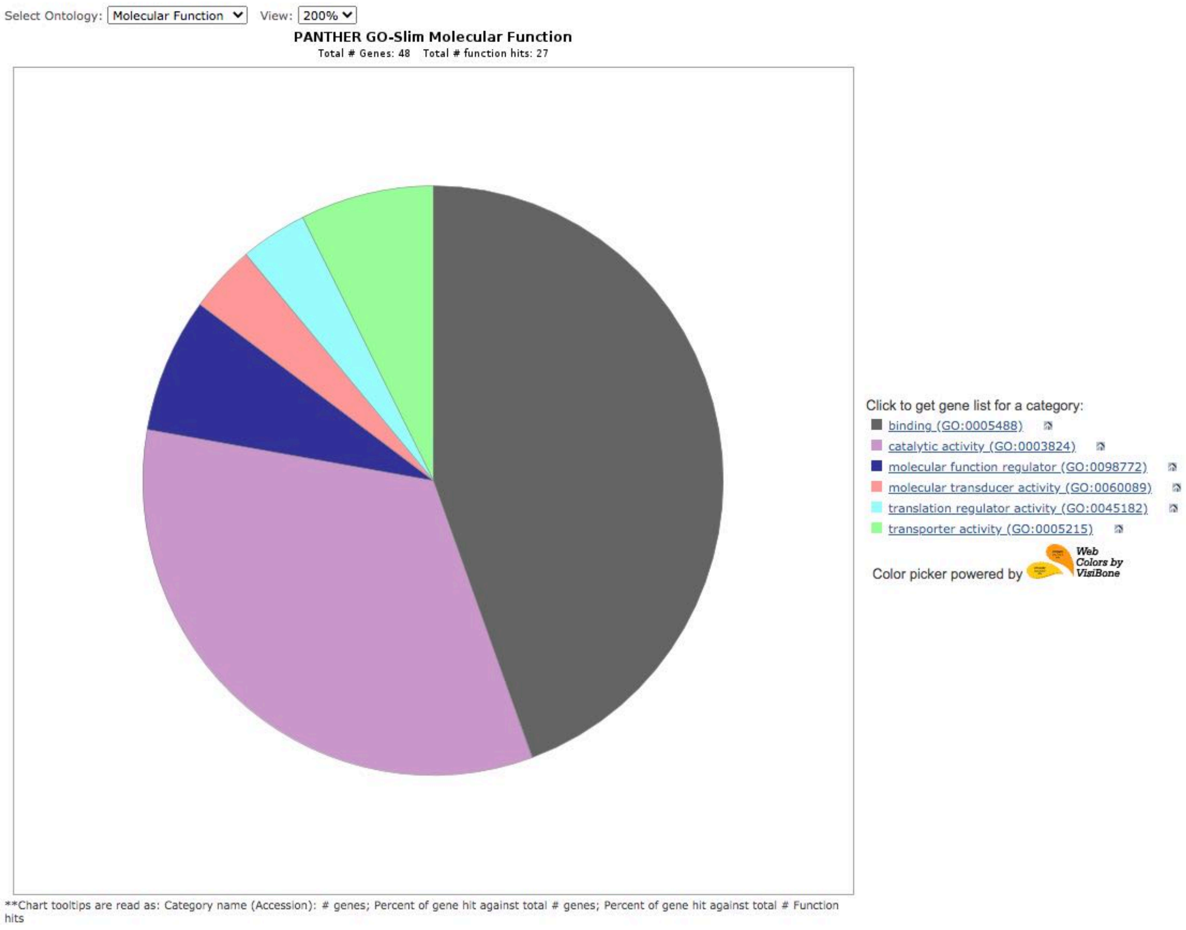

B

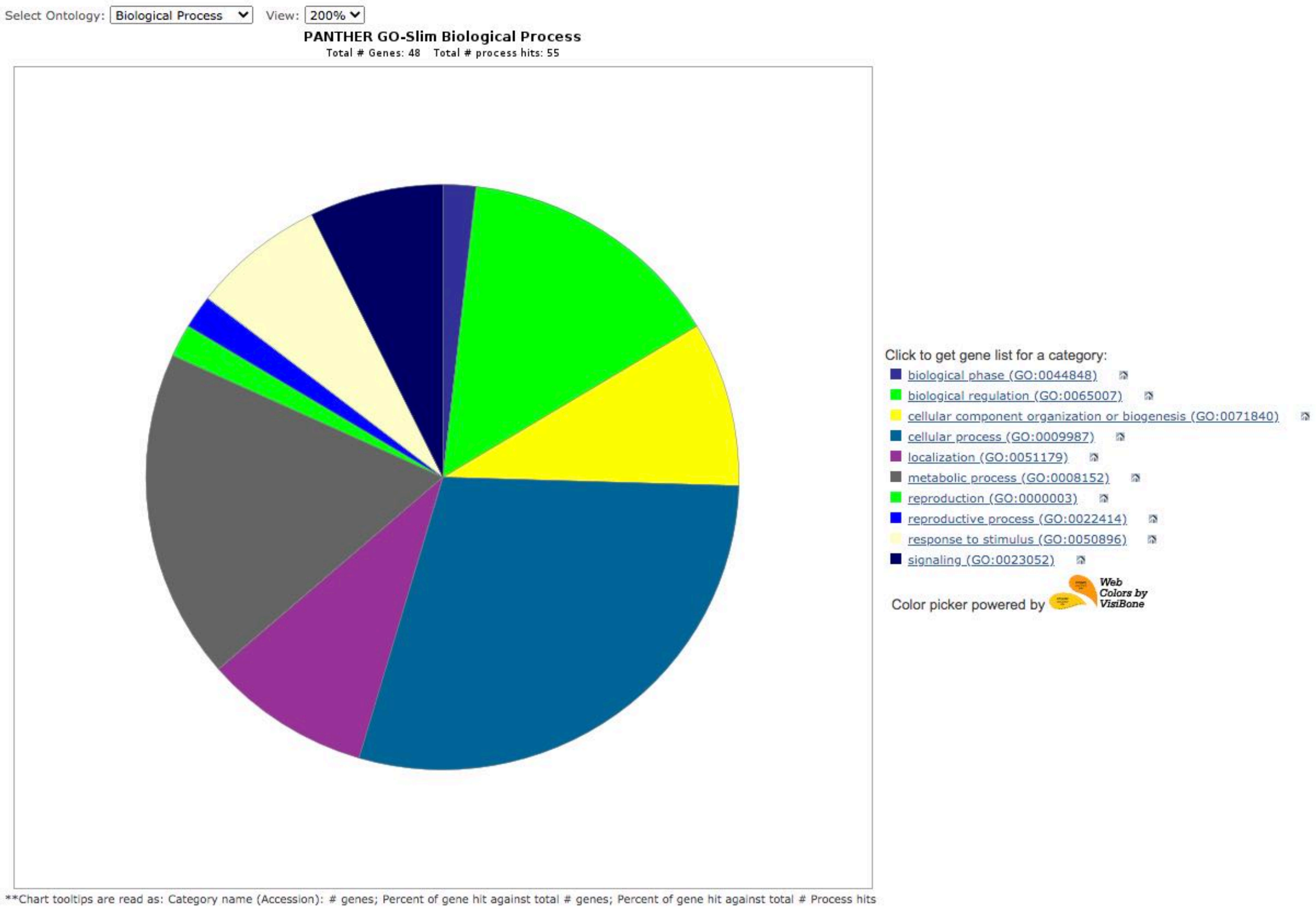

Supplementary Figure S4

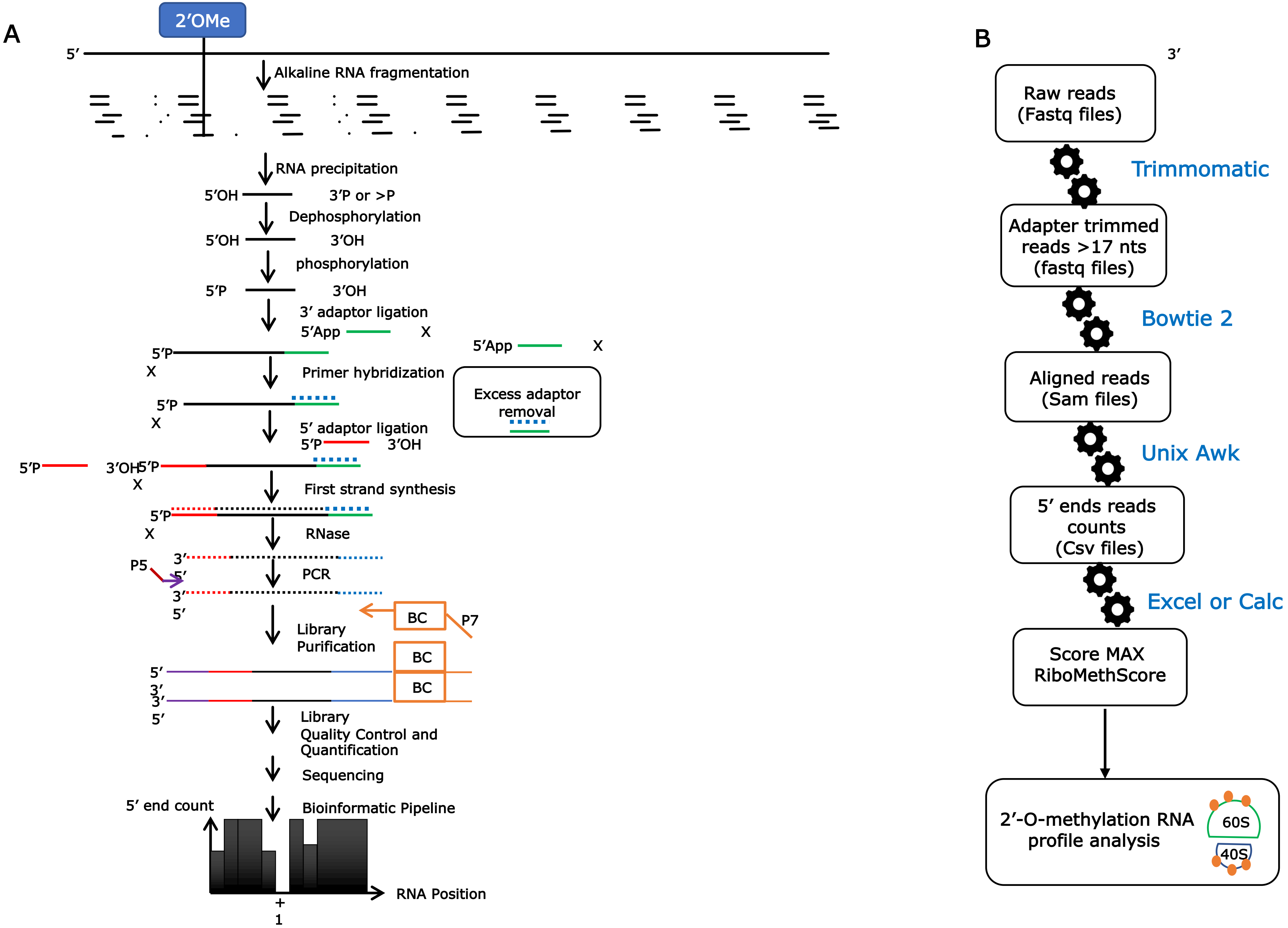

Supplement: Supplementary Figures 1–4 [file mmc4.pdf]
