# Supplementary material for: Ribosomal biogenesis regulator DIMT1 controls β-cell protein synthesis, mitochondrial function, and insulin secretion
Source: J Biol Chem. 2022 Feb 8;298(3):101692. doi: 10.1016/j.jbc.2022.101692 (PMC8913306; doi:10.1016/j.jbc.2022.101692)
Supplement: Supplementary Figures and Tables Captions [file mmc5.docx]

**Supplementary Figures**

**S1. Standardization and validation of DIMT1 knockdown**

Correlation of HbA1c and *DIMT1* gene expression (non-significant) is shown in (A). Protein levels of DIMT1 upon silencing with three different siRNAs was evaluated using western blot; actin was used as a loading control (B). UT-Untransfected, Sc-Scramble siRNA, 50 and 100 nM *DIMT1* siRNA. Data are expressed as mean ± SD (n=3) where **p <0.01. Protein synthesis was analyzed by OPP, using another set of siRNAs (SMARTpool), to ensure that effects observed in our study were caused by DIMT1-deficiency and not the siRNA2 sequence *per se* (c); compare graph in S1C with that in Figure 4D. Data are expressed as mean ± SD (n = 4), where **p <0.01 and where ***p <0.001. Cell viability was measured by counting the unstained cells with Trypan blue (D). Scramble siRNA-treated and *DIMT1* knockdown cells were stained with 0.4% of trypan blue post 72 h transfection. The graphical representation is shown of the trypan blue analysis and the data are expressed as mean ± SD (n=3). (E) Mitochondrial DNA level was evaluated by using qRT PCR for two mtDNA genes, *ND1* and *COX-1*, normalized with *GAPDH*. The data are shown as mean ± SD (n=3); the p-value was found to be not significant. NBR1 and DNAJC19 silencing at the protein level was determined by western blot in EndoC-βH1 cells (F); β-actin was used as a loading control. Data are expressed as mean ± SD (n=3) where **p <0.01 and ns is non-significant. (G) β-cell specific gene expression signatures were determined by qRT PCR. Four genes were tested to evaluate the effect of *DIMT1* knockdown on β-cell function. *PDX-1*, *LDHA*, *MAFA* and *INS-1* expression were normalized to actin. Statistical analysis was done using paired Student’s t-test and the data are mean ± SD (n=3) where *p <0.05, ns (non-significant).

**S2. Schematic representation of primer extension assay**

Hemo KlenTaq is a truncated version of Taq DNA Polymerase that allows quantification of methylation in the sample. Two different primers (sense and anti-sense) were designed flanking the two methylated adenosine residues (1850 and 1851) on human 18s rRNA. Hemo KlenTaq polymerase was used, with reverse transcriptase activity but lacking processivity blocked by the presence of RNA methylation. The relative gene expression of control and DIMT1 knockdown was calculated by the ratio between the target and the reference gene.

**S3. PANTHER Pathways database analysis of significantly altered genes**

A PANTHER pathway database analysis was performed with a total of 48 significantly expressed genes, which showed significant group differences; this identified 27 molecular functional pathways S3 (A) and 55 biological processes hits S3 (B). The topmost pathways shown emerged from binding, catalytic activity and translation regulator in molecular function S3 (A), whereas the maximum hits in biological processes were from biological phase, regulation, cellular biogenesis, localization and metabolic process S3 (B). For the full index lists of the genes by Ribomethseq from which the 48 significantly altered genes were selected for Panther pathway categories, see Supplementary Table S3.

**S4. Schematic representation of experimental and statistical description of RNA sequencing**

(A). General overview of the RiboMethSeq method. Control and DIMT1 knockdown RNA containing 2′-O- Me residues were randomly fragmented by the alkaline hydrolysis method. 5′- and 3′-ends were repaired and adapters were ligated to both the ends. Post amplification and barcoding, amplicons are subjected to the Illumina sequencing platform on HiSeq1000 in single-read mode. The 2′-O-Me modification prevents the 3′ phosphodiester bond from cleavage, generating a typical gap in 5′-ends. (B). Bioinformatics pipeline for the RiboMethSeq analysis. Raw reads after sequencing experiments are trimmed using Trimmomatic to remove adapter sequences, aligned to the reference sequence by Bowtie2 and 5′-ends of the reads were evaluated. Calculations of ScoreMAX and RiboMethScore were performed using the DSeq2 and Salmon platform. The schematic of the RNA sequencing protocol and analysis were adapted from (ebook ISBN 978-1-4939-6807-7, chapter-12).

**Supplementary Tables:**

**Table S1.** Multiple nominal eQTLs signals for SNPs mapping to the *DIMT1* locus in the islets are shown. Look-up in previous GWAS data from the T2D Diabetes Knowledge Portal showed the same SNPs to be significantly associated with blood pressure and a second suggestive signal associated with BMI and two-hour insulin values and additional glycemic traits.

**Table S2.** RNA sequencing data revealed methylated sites on 5S and 28S. Listed are the methylated sites in scramble control and cells treated with *DIMT1* siRNA.

**Table S3.** Differentially expressed genes determined by RNA sequencing are listed. The log2 fold change for the cells treated with *DIMT1* knockdown are plotted with an adjusted p-value below a threshold (here 0.1, the default) are shown. The normalized average mean counts in the differentially expressed genes are shown in the left (green) panel in the tab counts whereas the cells treated with *DIMT1* siRNA are shown in the right panel (orange).
